# Supplementary material for: Topologically guided tuning of Zr-MOF pore structures for highly selective separation of C6 alkane isomers
Source: Nat Commun. 2018 May 1;9:1745. doi: 10.1038/s41467-018-04152-5 (PMC5931593; doi:10.1038/s41467-018-04152-5)
Supplement: Supplementary file 1 — Supplementary Information [file 41467_2018_4152_MOESM1_ESM.pdf]

Supplementary Information

Topologically Guided Tuning of Zr-MOF Pore Structures for Highly Selective Separation of C6 Alkane Isomers

Wang et al.

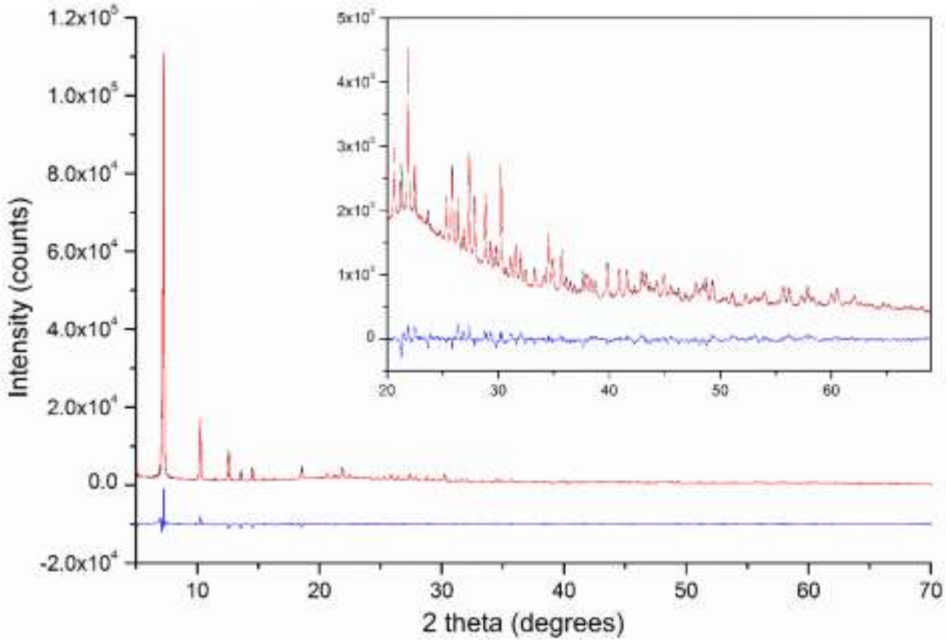

Supplementary Figure 1 │ Observed (black), calculated (red) and difference (blue) PXRD profiles for the activated Compound **1** based on the Rietveld refinement results. It was revealed that compound **1** crystalizes in a cubic space group Im-3 (*a* = 24.3597 (3) Å), through the Rietveld refinement against the Powder X-ray diffraction pattern. The position of Zr-Cluster was firstly solved by direct method (EXPO[1]) using the Powder X-ray diffraction pattern. Then, the location of organic linker was determined using simulated annealing method by TOPAS[2] in the space group of I23. An initial model was successfully built up and the symmetry was further increased into Im-3 later. At last, Rietveld refinement was made based on the model obtained above, to achieve the final structure model of this Zr-MOF material.

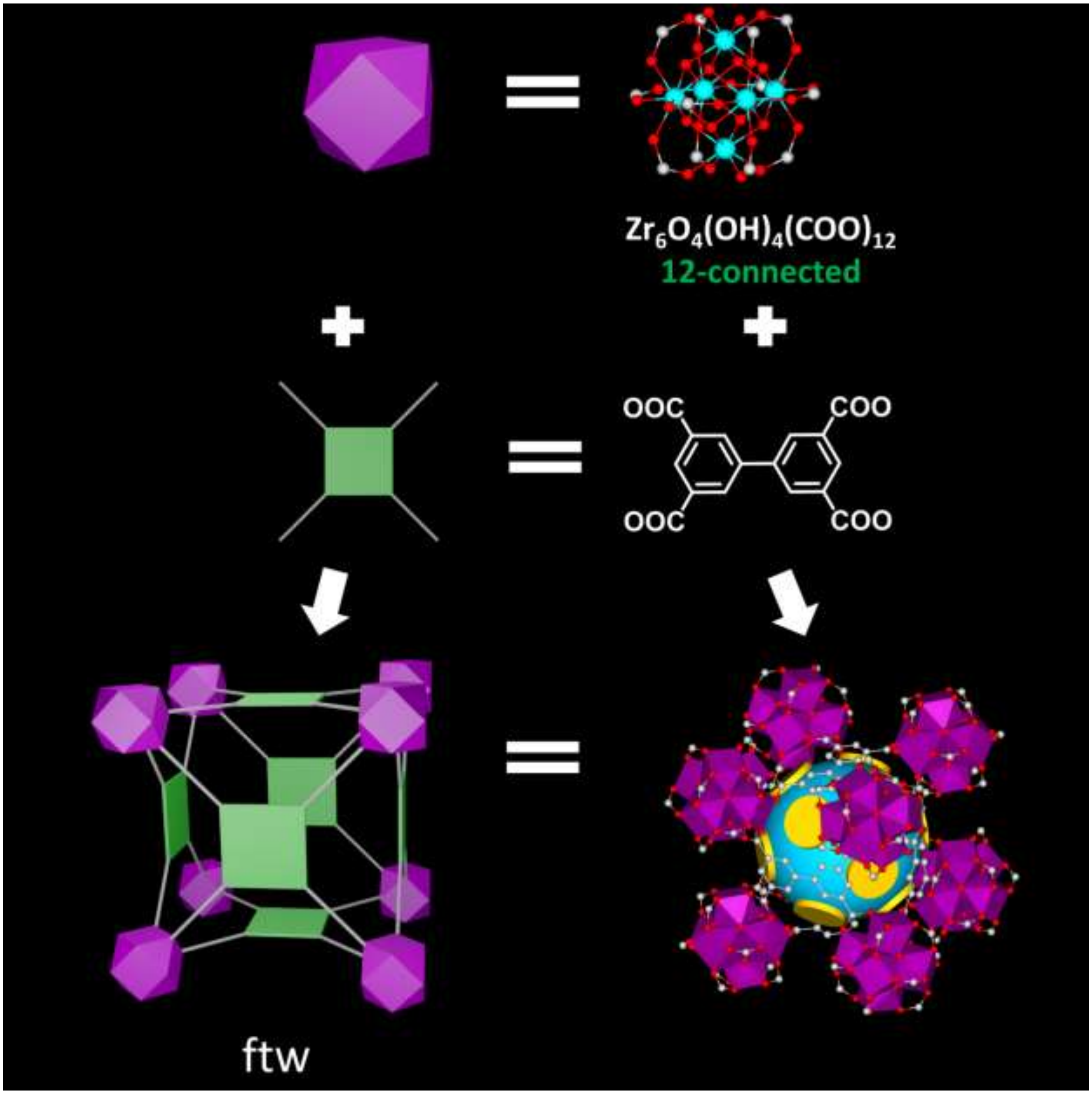

Supplementary Figure 2 │ Crystal structure and topology of compound **1**.

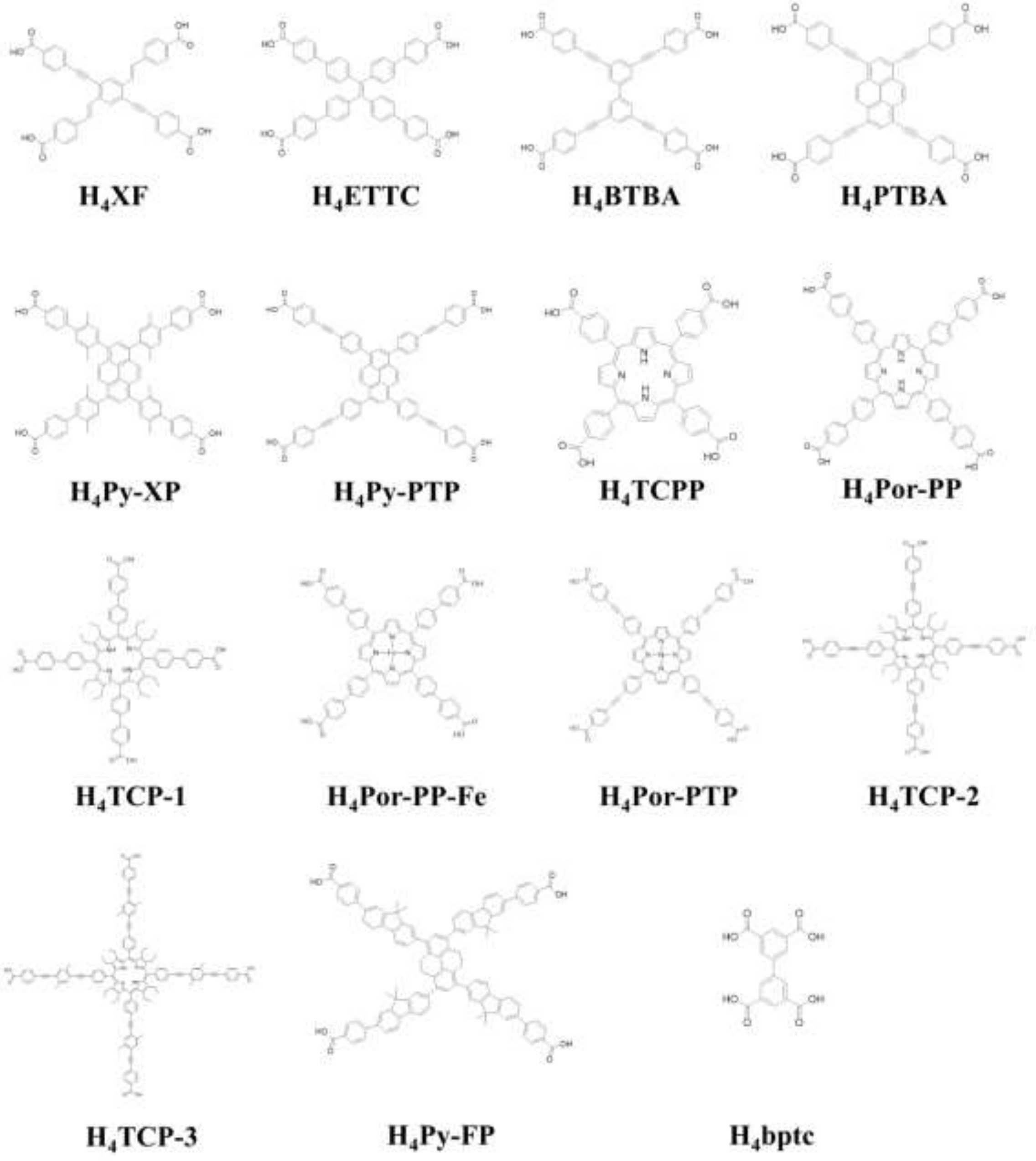

Supplementary Figure 3 | Organic linkers used to construct **ftw** Zr-MOFs.

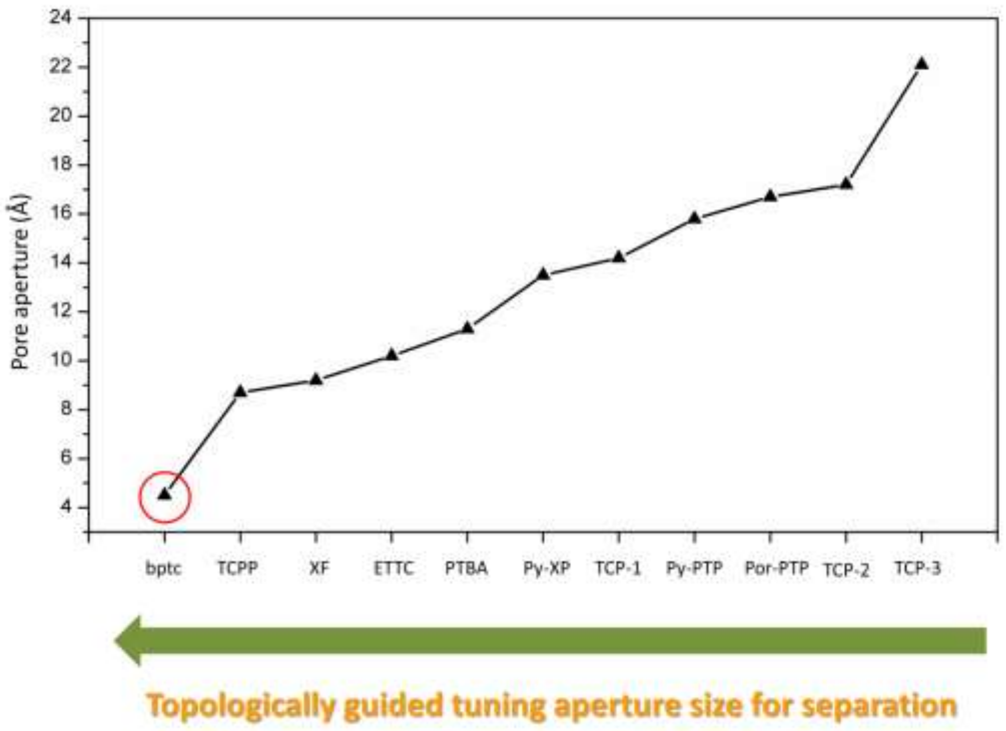

Supplementary Figure 4 | Pore aperture of **ftw** Zr-MOFs built on different linkers. (The aperture size was calculated from the shortest distance connecting the opposite sides of the pore window which exclude van der Waals radii (vdW radii used: C: 1.7 Å, H: 1.20 Å, O: 1.52 Å)).

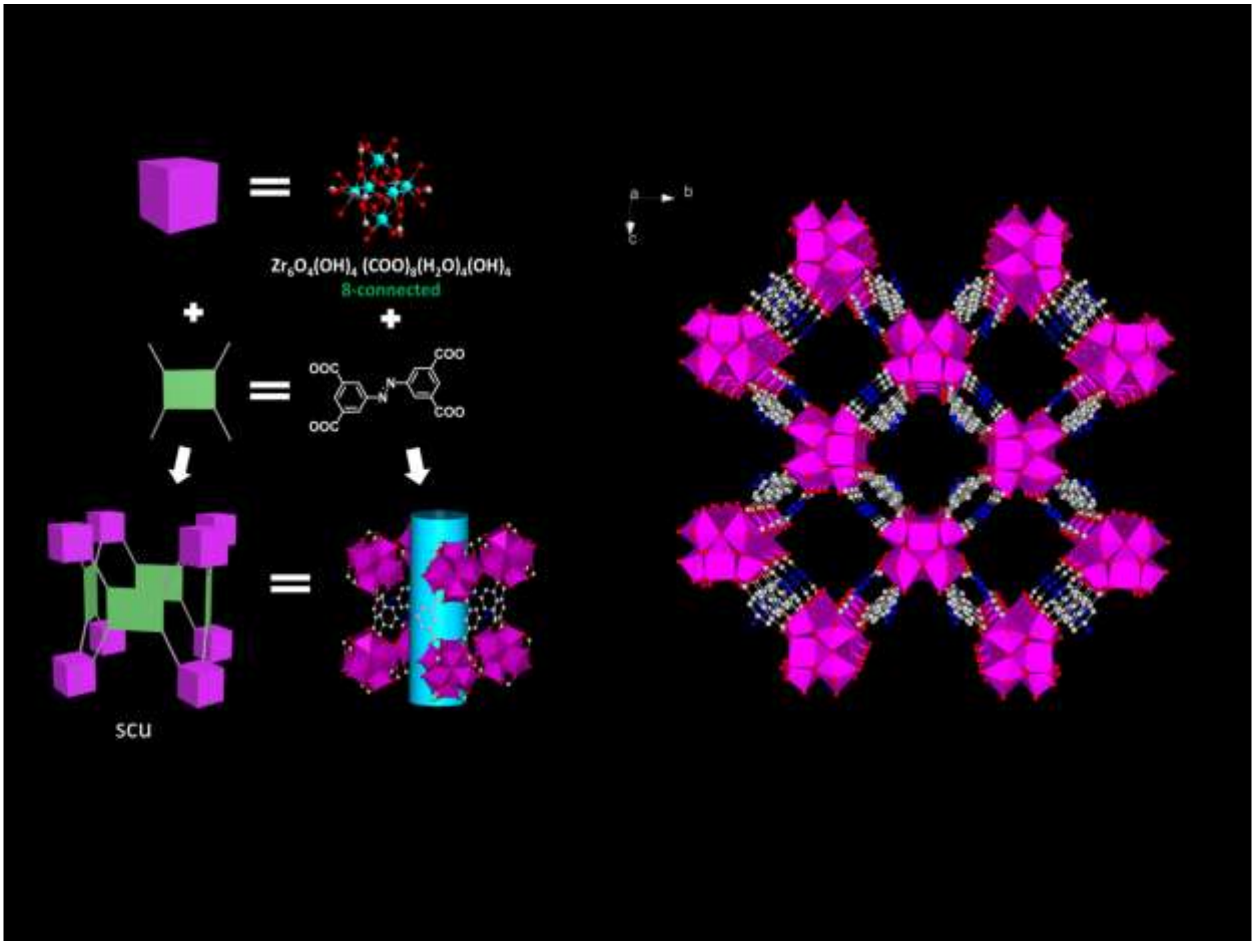

Supplementary Figure 5 │ Crystal structure and topology of compound **2**.

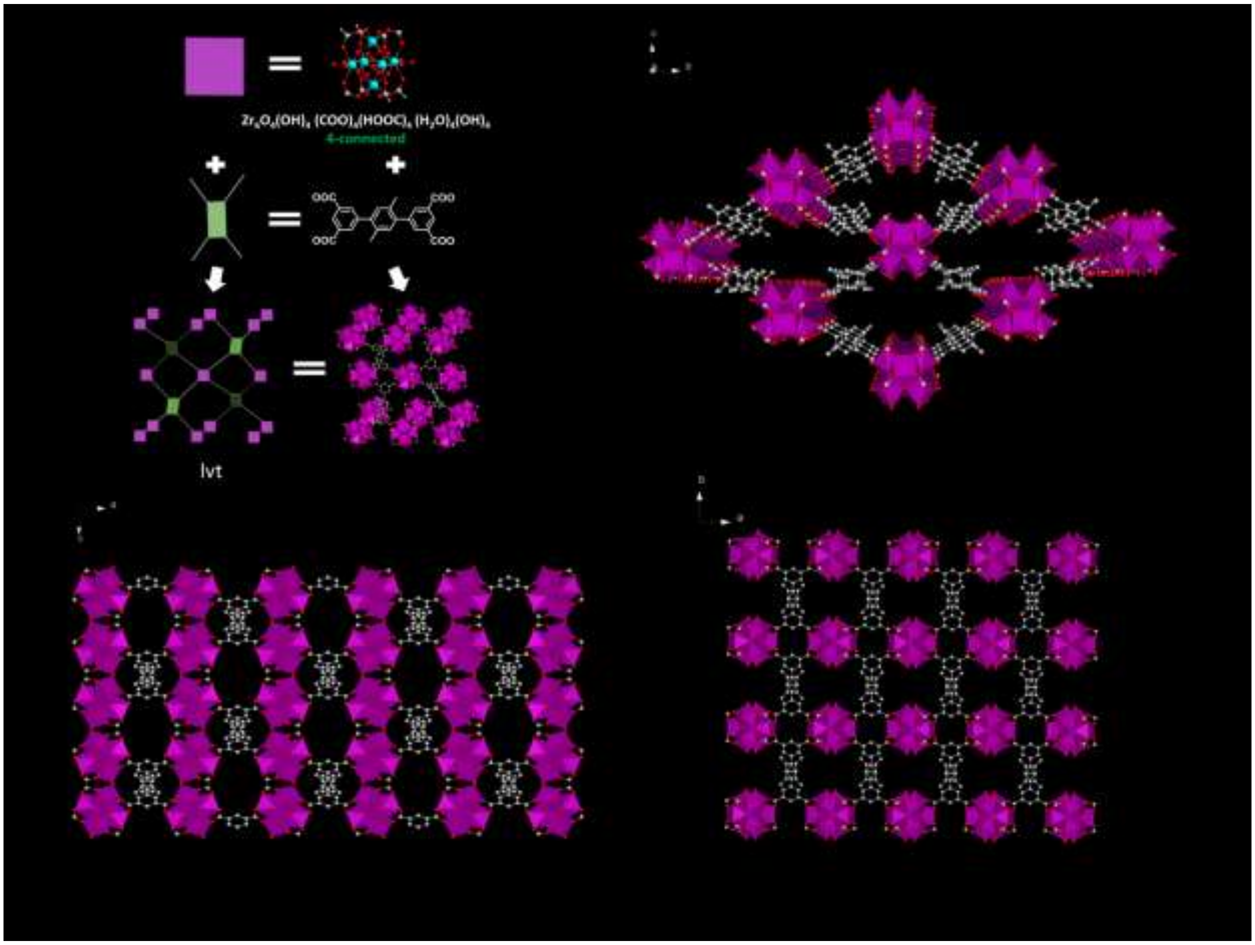

Supplementary Figure 6 │ Crystal structure and topology of compound **3**.

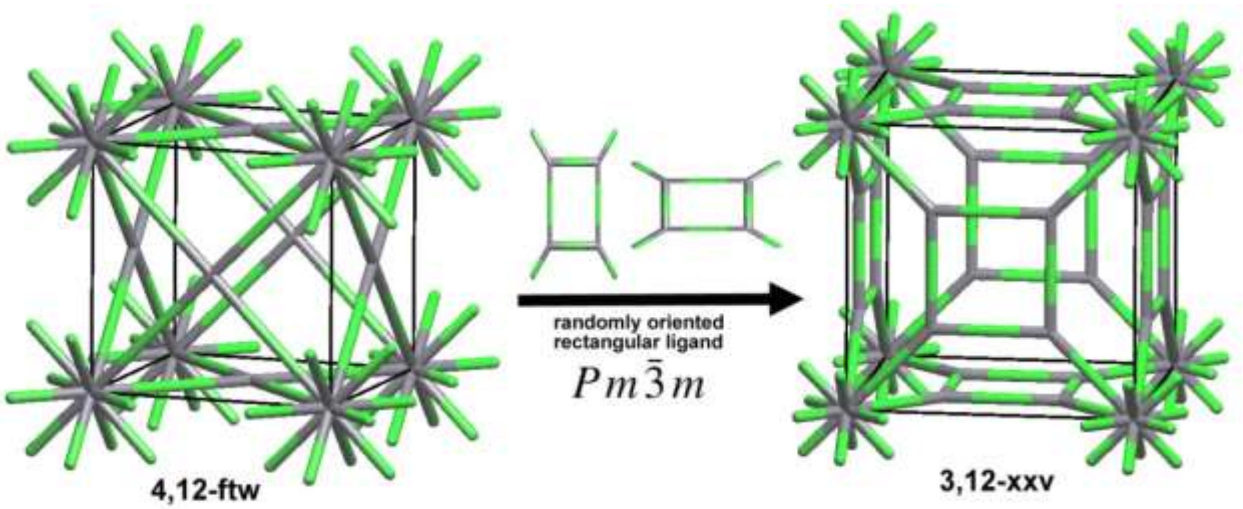

Supplementary Figure 7 │ Two possible relative orientations of a rectangular ligand in 4,12-**ftw** topology.

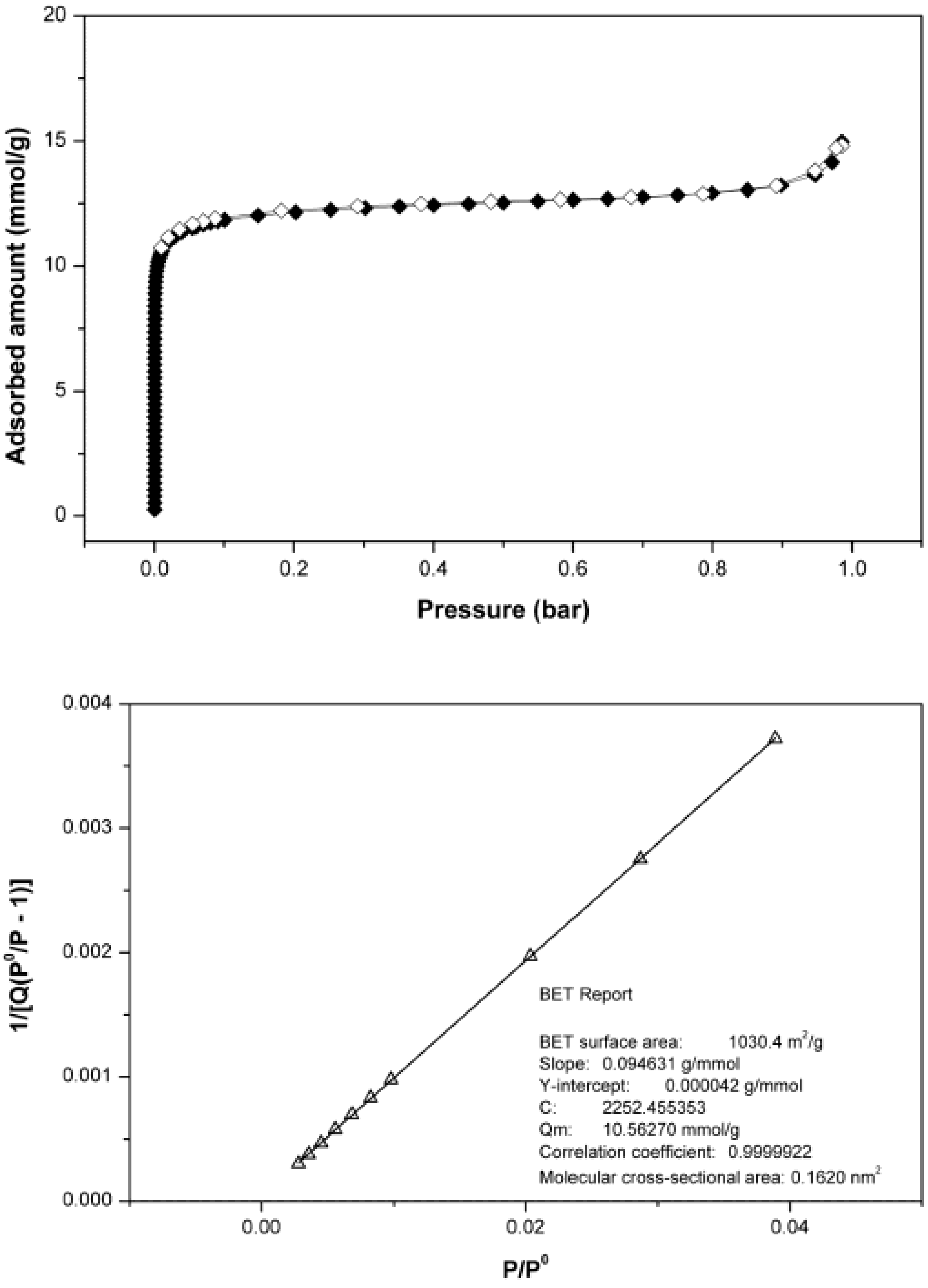

Supplementary Figure 8 │ N_2 adsorption isotherm at 77 K (top) and BET plot (bottom) for compound **1**.

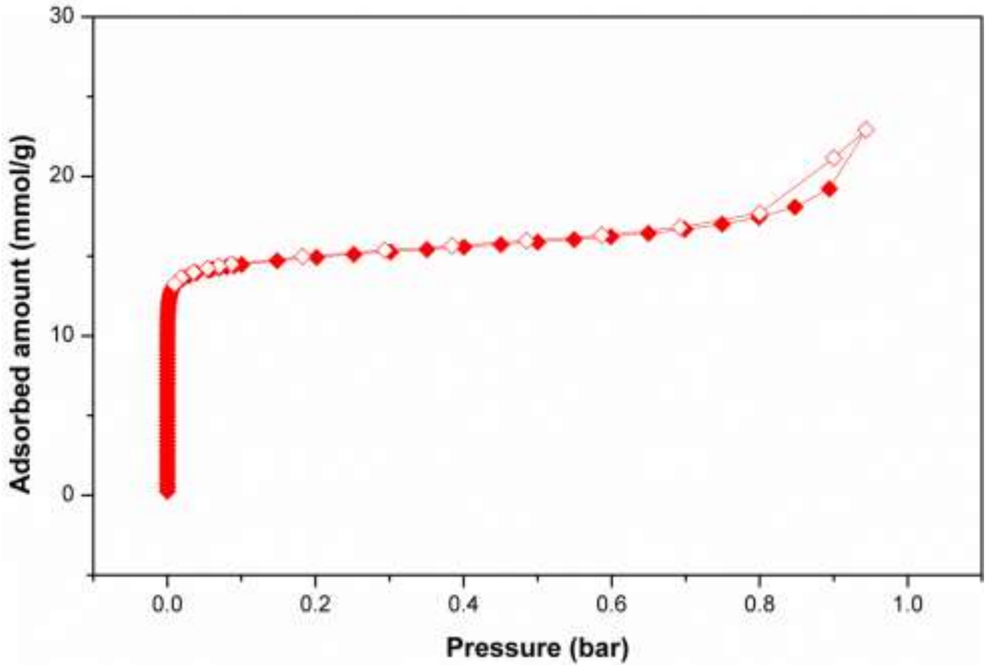

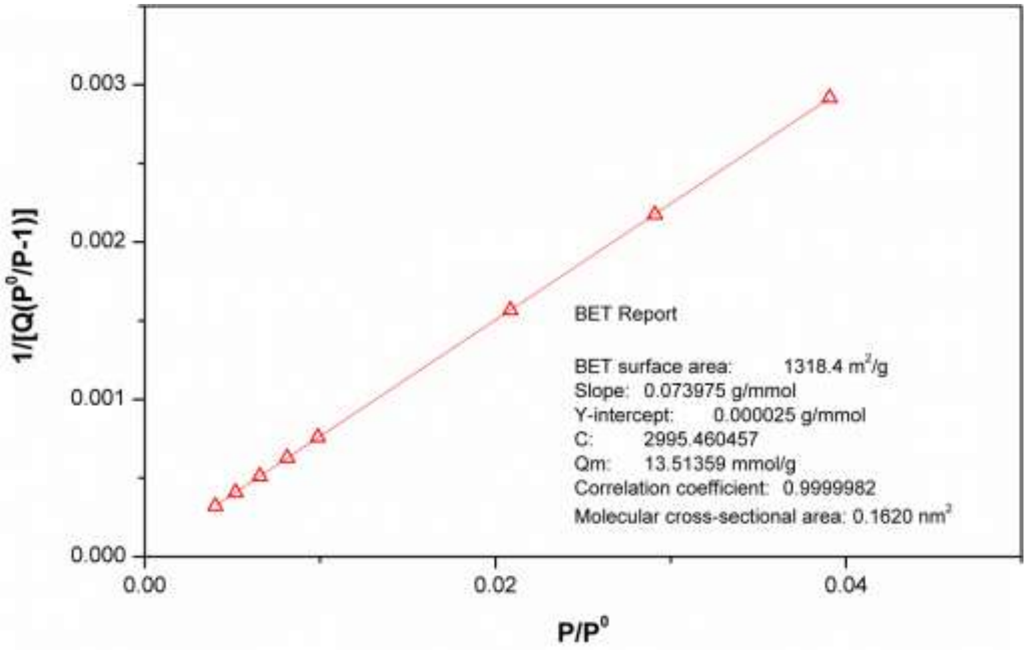

Supplementary Figure 9 │ N_2 adsorption isotherm at 77 K (top) and BET plot (bottom) for compound **2**.

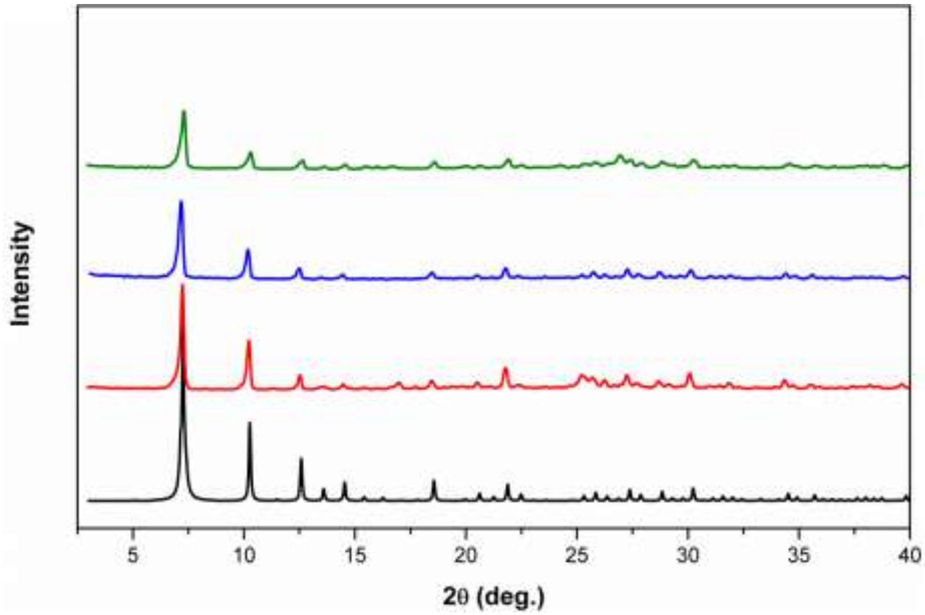

Supplementary Figure 10 │ PXRD patterns of compound **1**. From bottom to top: simulated (black), as synthesized (red), activated (blue), after adsorption study (green).

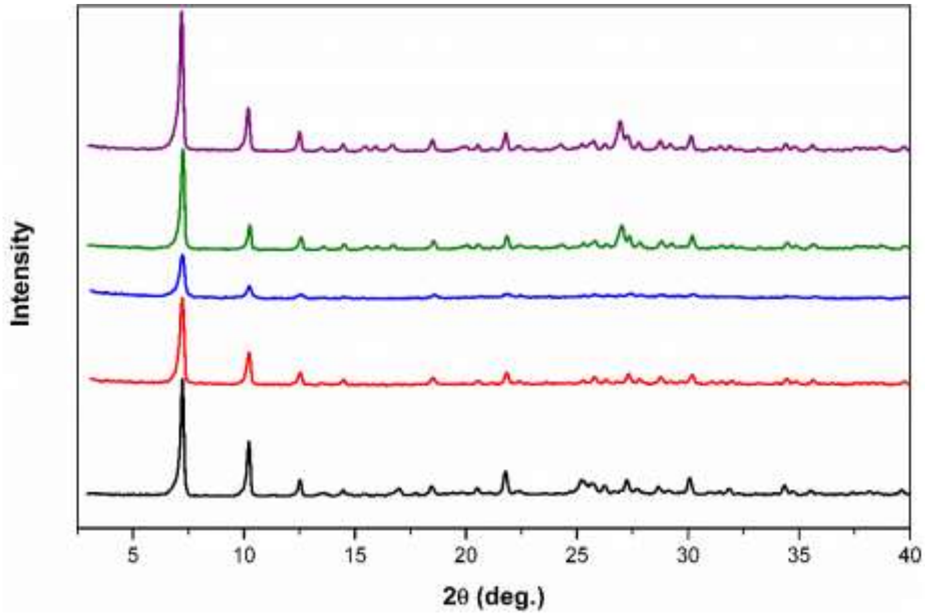

Supplementary Figure 11 | PXRD patterns of compound **1**. From bottom to top: as synthesized (black), 300 °C isothermal for 2 hours in nitrogen (red), 400 °C isothermal for 2 hours in nitrogen (blue), 180 °C in open air for 1 week (green), 180 °C in open air for 1 month (purple).

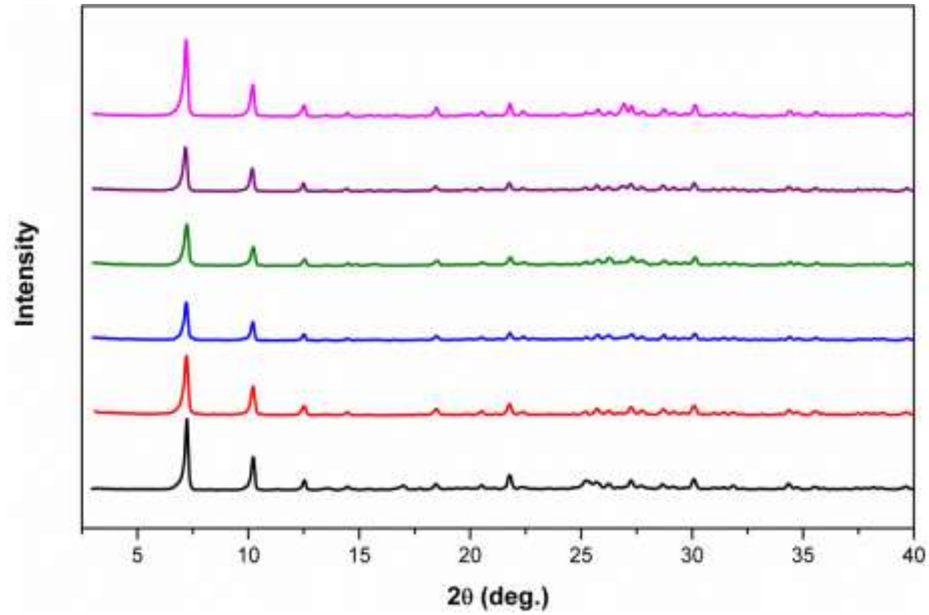

Supplementary Figure 12 | ** PXRD patterns of compound **1. From bottom to top: as synthesized (black), immersed in water for 1 week (red), immersed in pH = 2 aqueous HCl solution for 1 week (blue), immersed in pH = 12 NaOH aqueous solution for 1 week (green), immersed in 100 °C water (in teflon bomb) for 1 week (purple), immersed in hexane isomers solution (*n*-hexane: 3-methylpentane: 2,2-dimethylbutane= 1:1:1) for 1 month (magenta).

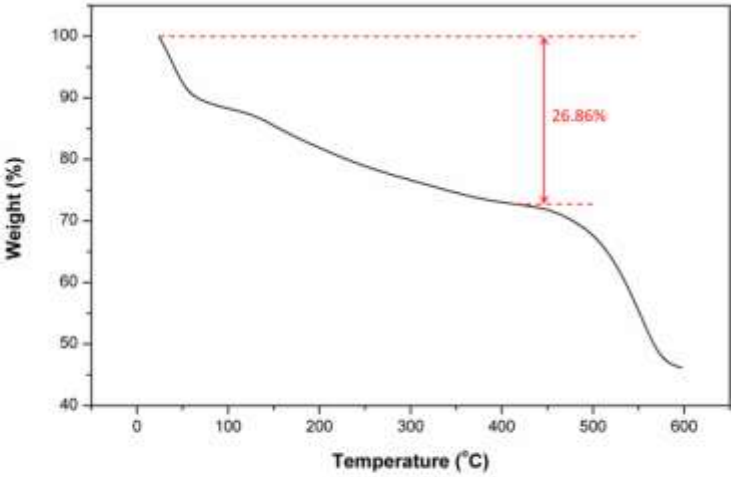

Supplementary Figure 13 │ TGA curve of compound **1**.

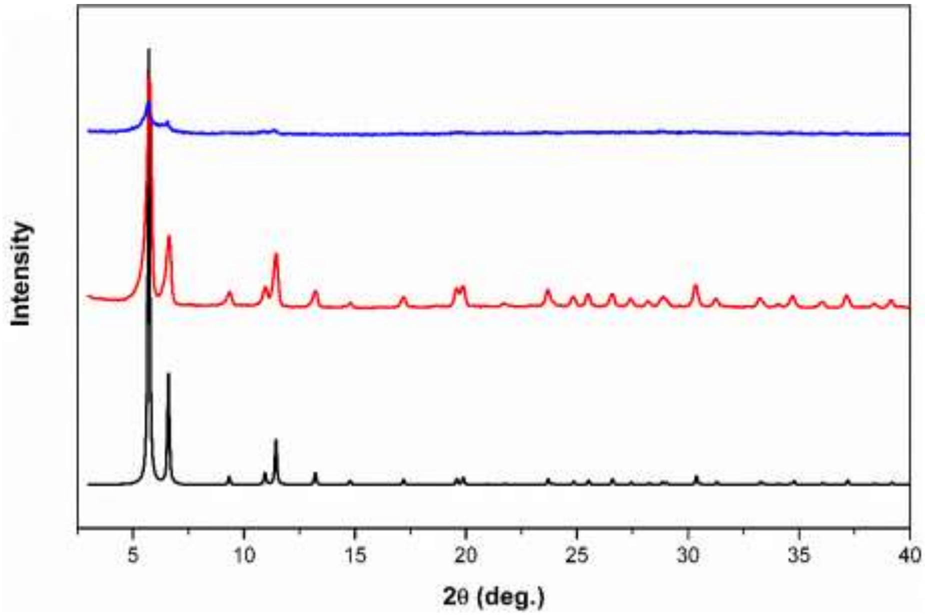

Supplementary Figure 14 │ PXRD patterns of UiO-67: Simulated (black), as synthesized (red) and after immersed in H_2O at 80 °C for 1 day.

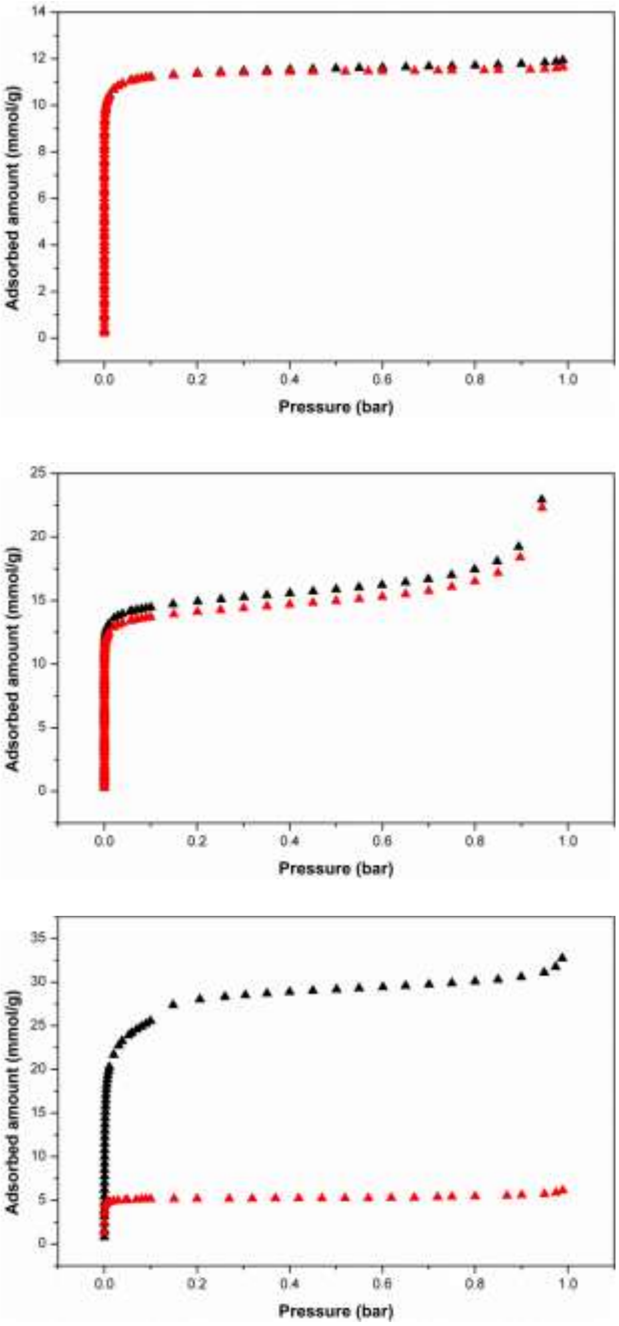

Supplementary Figure 15 │ N_2 adsorption isotherms at 77 K before (black) and after (red) water treatment for compound **1** (top), compound **2** (middle) and UiO-67 (bottom). (Water treatment: ~100 mg of sample immersed in ~20 mL of water which is heated at 80 °C for 24 hours).

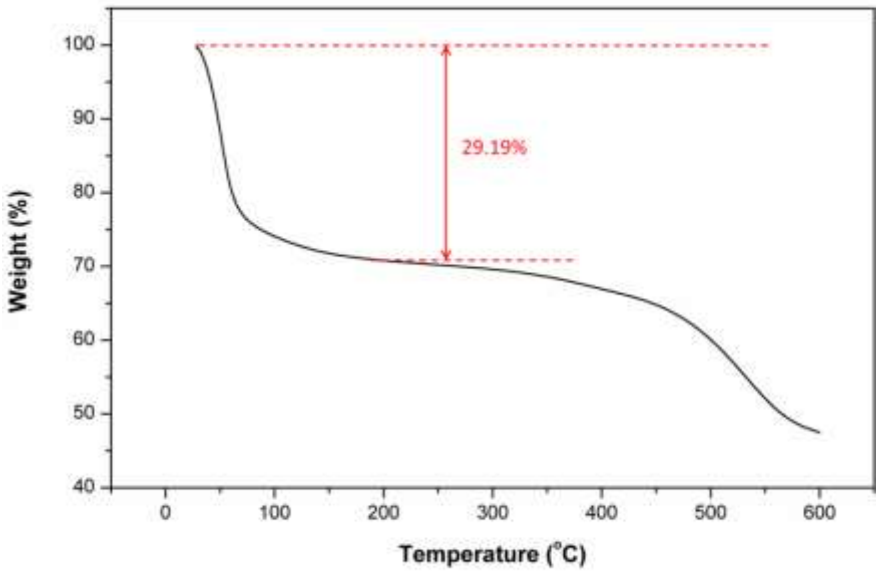

Supplementary Figure 16 │ TGA curve of compound **2**.

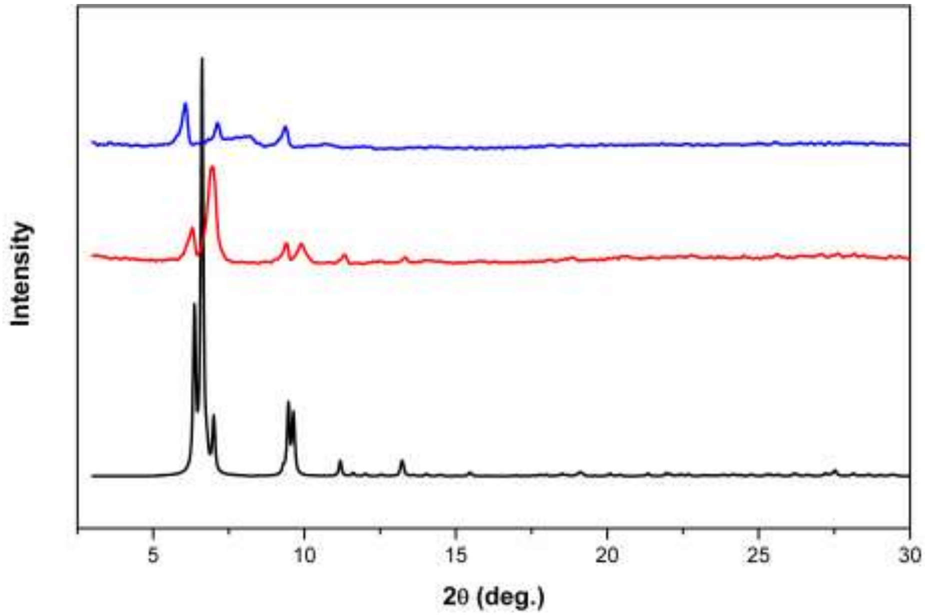

Supplementary Figure 17 | ** PXRD patterns of compound **3. From bottom to top: simulated (black), as synthesized (red), activated (blue).

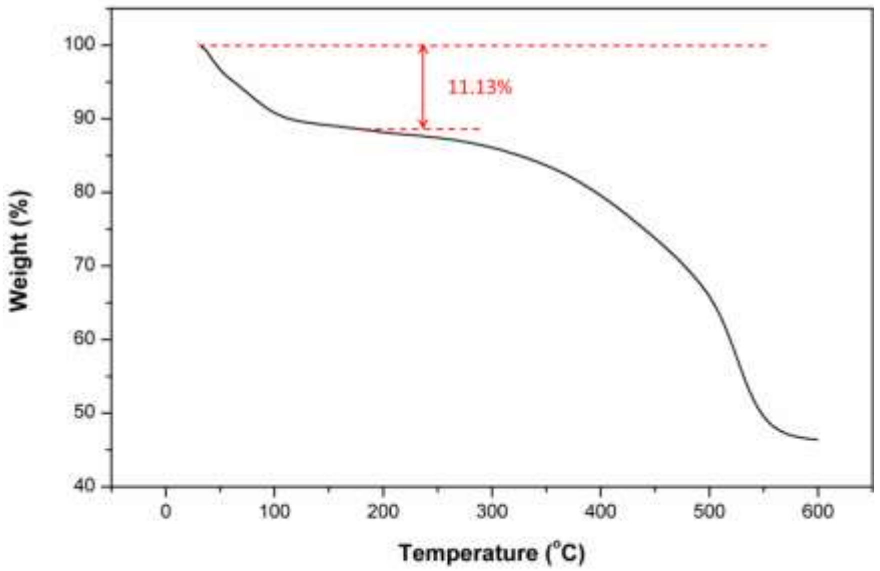

Supplementary Figure 18 │ TGA curve of compound **3**.

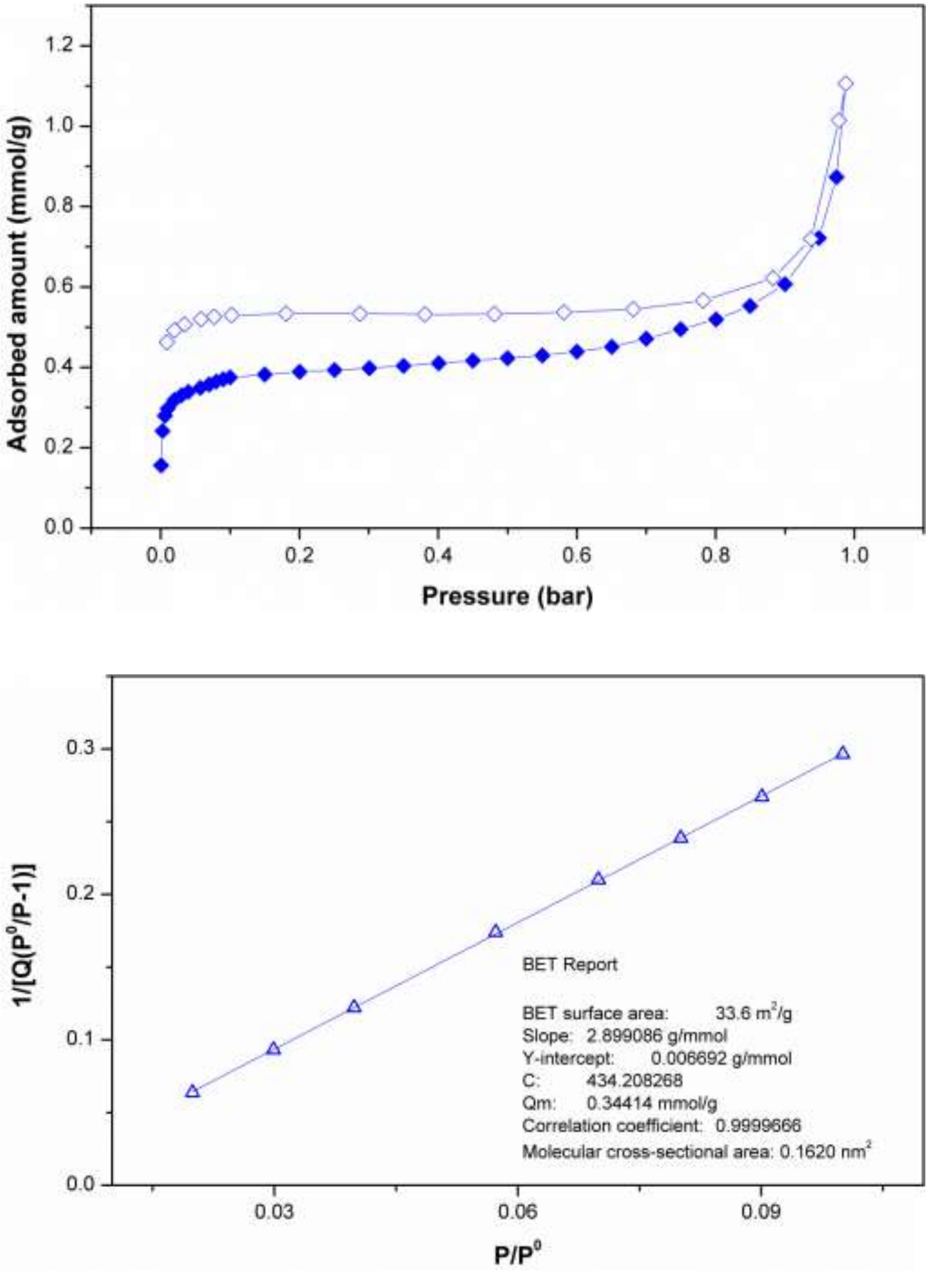

Supplementary Figure 19 │ N_2 adsorption isotherm at 77 K (top) and BET plot (bottom) for compound **3**. The measured BET surface area (33.6 m^2/g) is much lower than its theoretical value (2500 m^2/g), calculated based on its crystal structure.

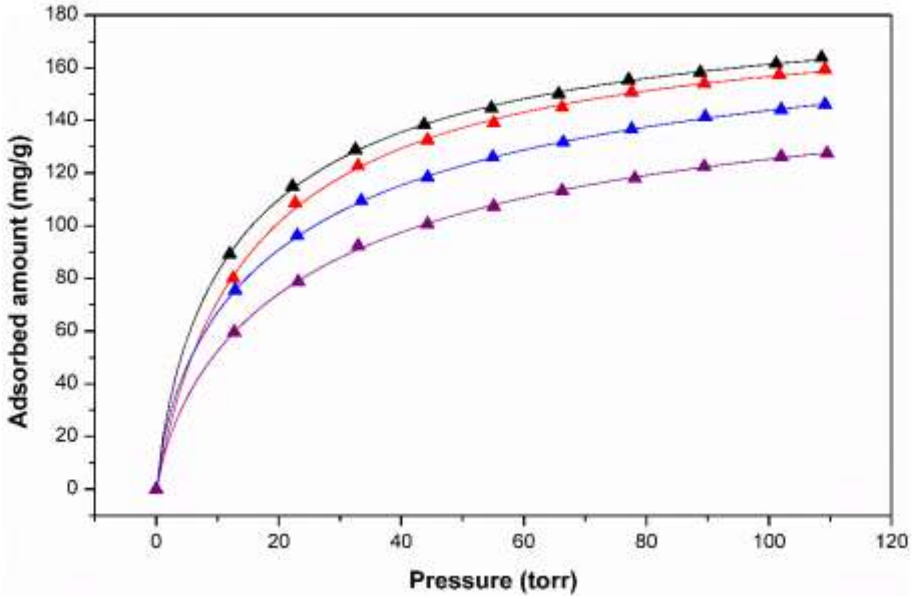

Supplementary Figure 20 │ *n*-hexane adsorption isotherms on compound **1** at 30 °C (black), 90 °C (red), 120 °C (blue) and 150 °C (purple).

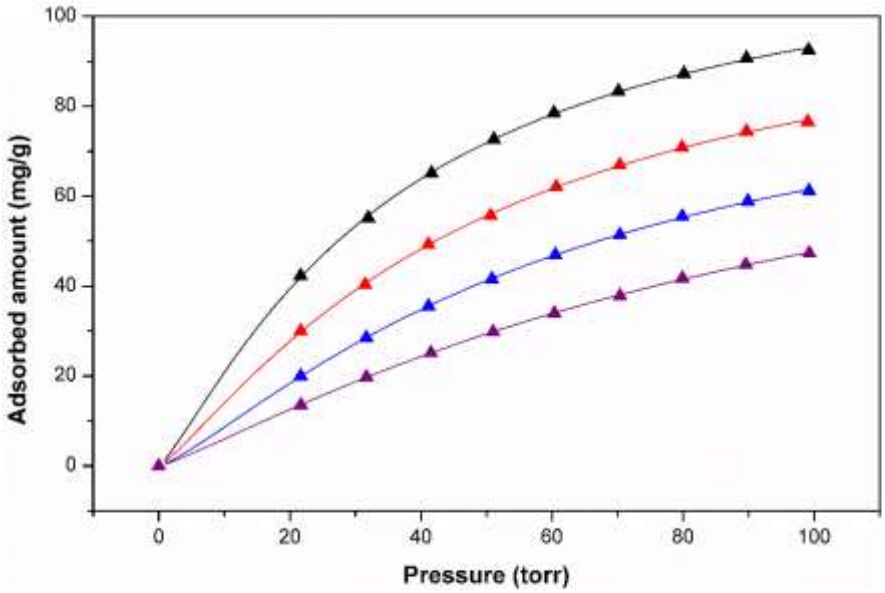

Supplementary Figure 21 │ *n*-hexane adsorption isotherms on compound **1** at 180 °C (black), 200 °C (red), 220 °C (blue), and 240 °C (purple).

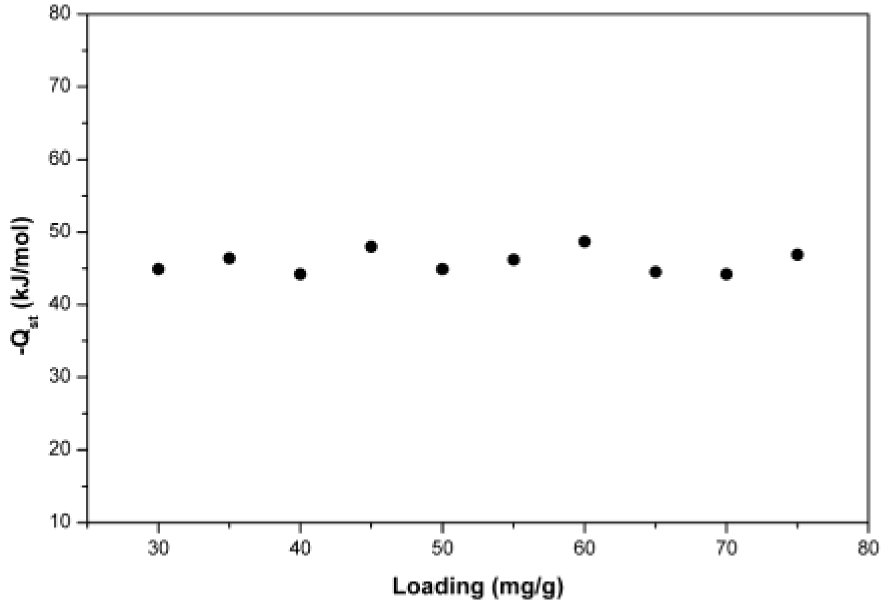

Supplementary Figure 22 │ Isosteric heats of adsorption (Q_{st}) for *n*-hexane adsorption on compound **1**.

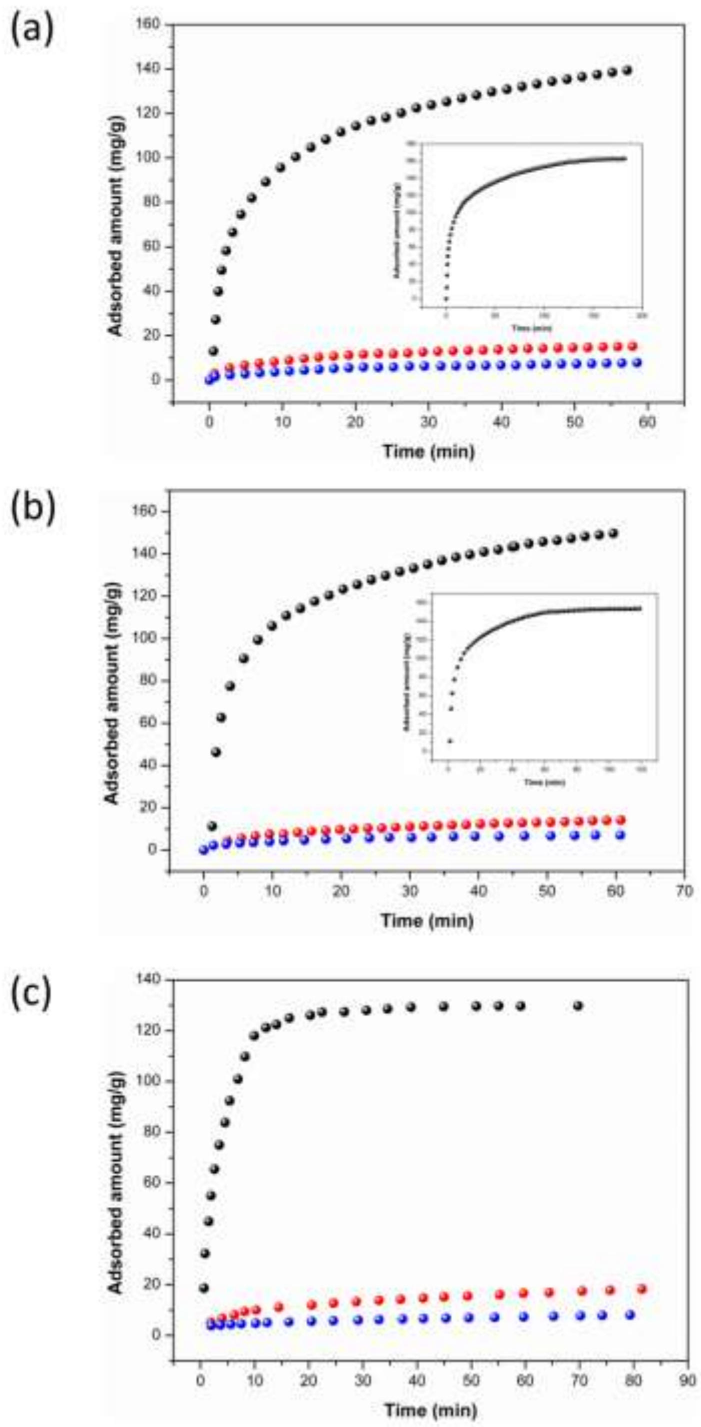

Supplementary Figure 23 │ Adsorption of *n*-hexane (black), 3-methylpentane (red) and 2,3-dimethylbutane (blue) on compound **1** at (a) 30 °C (top), (b) 90 °C (middle), and (c) 150 °C (bottom).

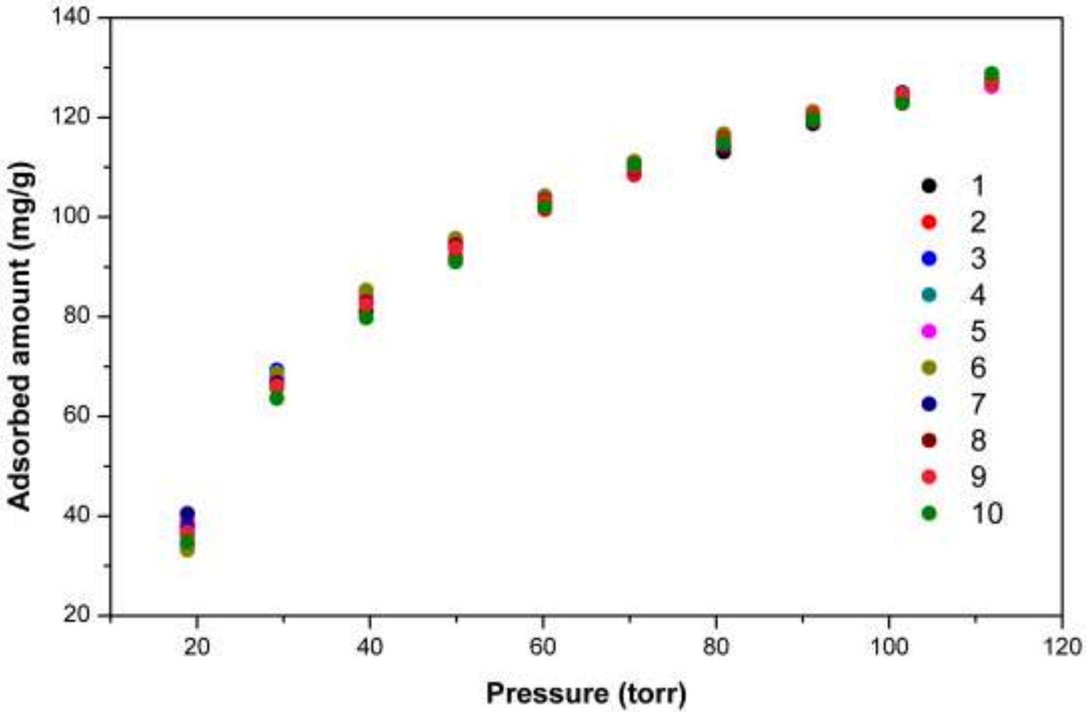

Supplementary Figure 24 │ Ten cycles of *n*-hexane adsorption isotherms on compound **1** at 150 °C (desorption condition: 150 °C for 30 min under nitrogen).

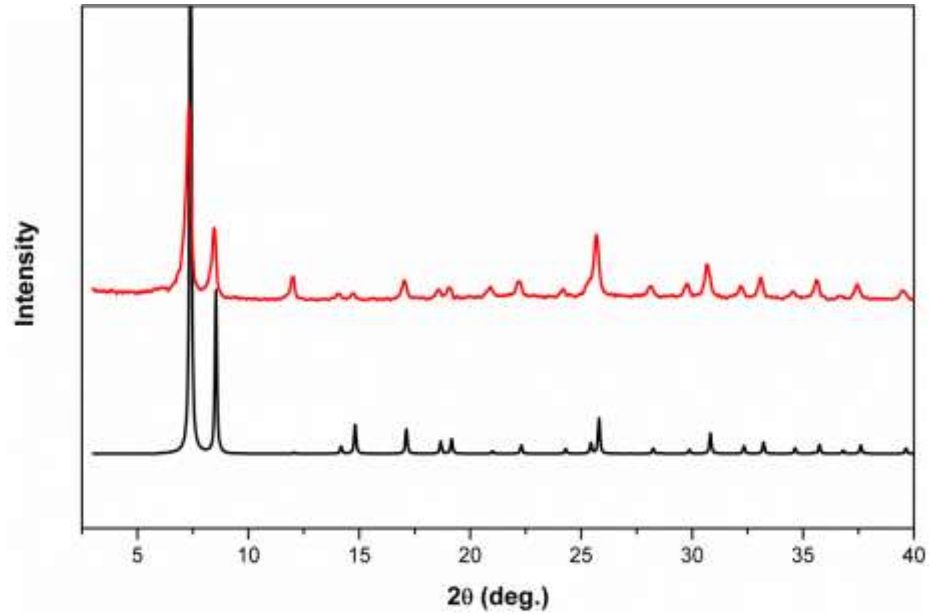

Supplementary Figure 25 | PXRD patterns of UiO-66. Simulated (black), as synthesized (red).

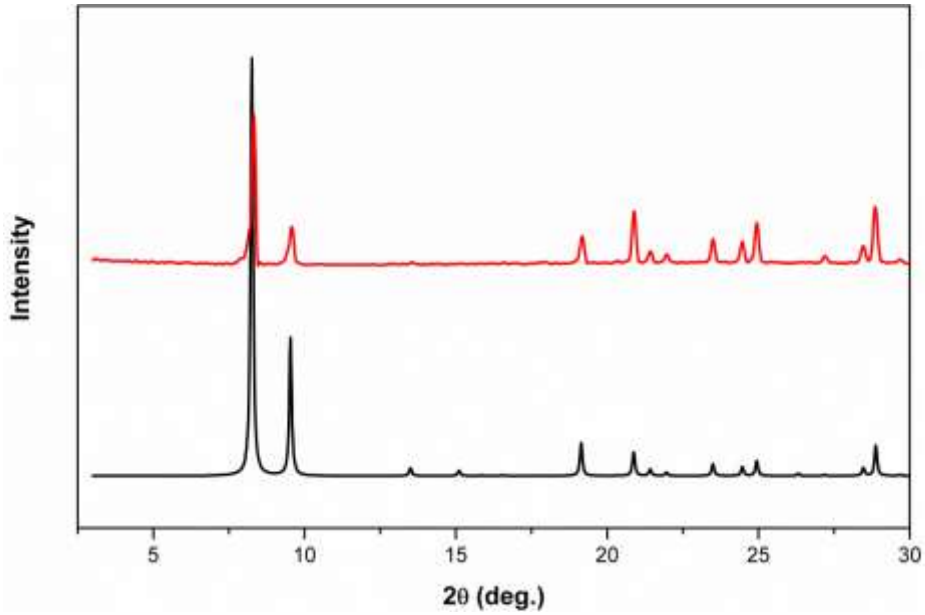

Supplementary Figure 26 │ PXRD patterns of Y-fum. Simulated (black), as synthesized (red).

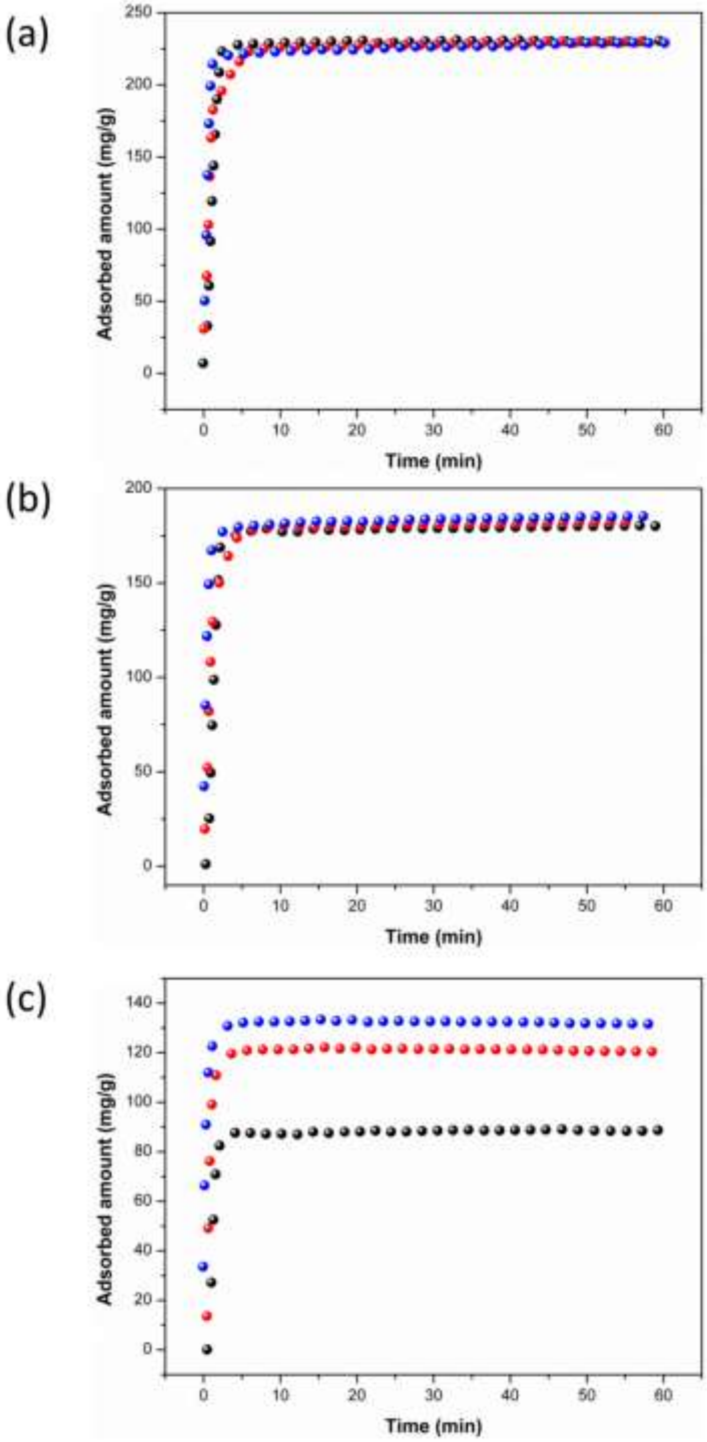

Supplementary Figure 27 | Adsorption of *n*-hexane (black), 3-methylpentane (red) and 2,3-dimethylbutane (blue) on UiO-66 at (a) 30 °C, (b) 90 °C, and (c) 150°C.

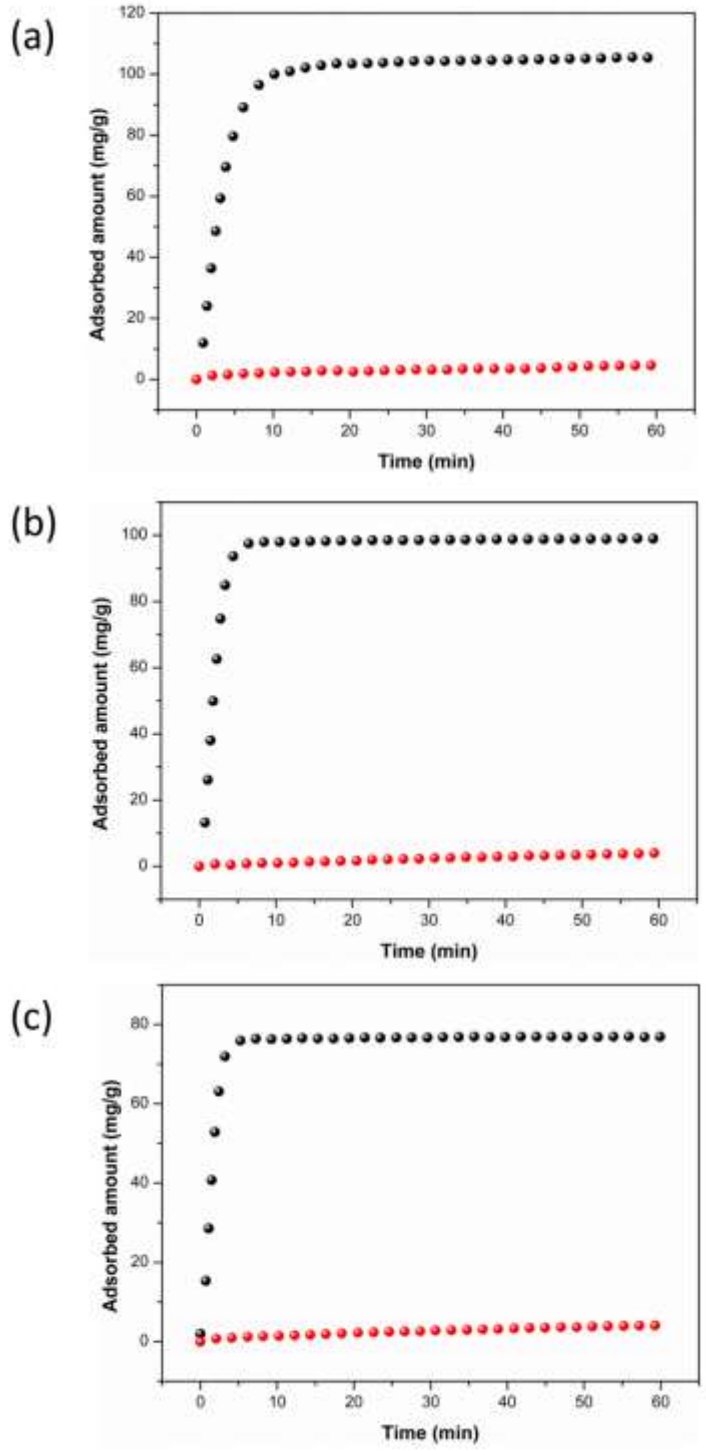

Supplementary Figure 28 │ Adsorption of *n*-hexane (black) and 3-methylpentane (red) on zeolite 5A at (a) 30 °C, (b) 90 °C, and (c) 150°C.

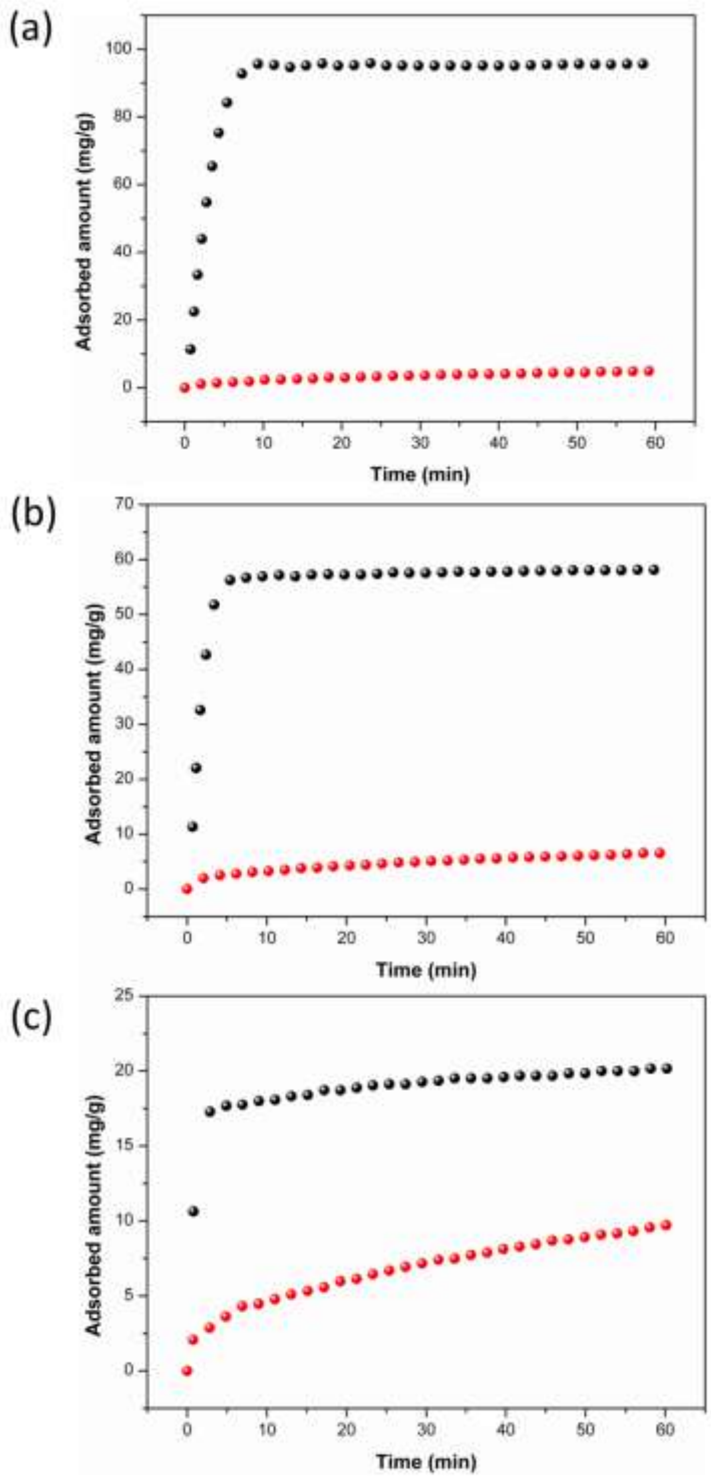

Supplementary Figure 29 │ Adsorption of *n*-hexane (black) and 3-methylpentane (red) on Y-fum at (a) 30 °C, (b) 90 °C, and (c) 150°C.

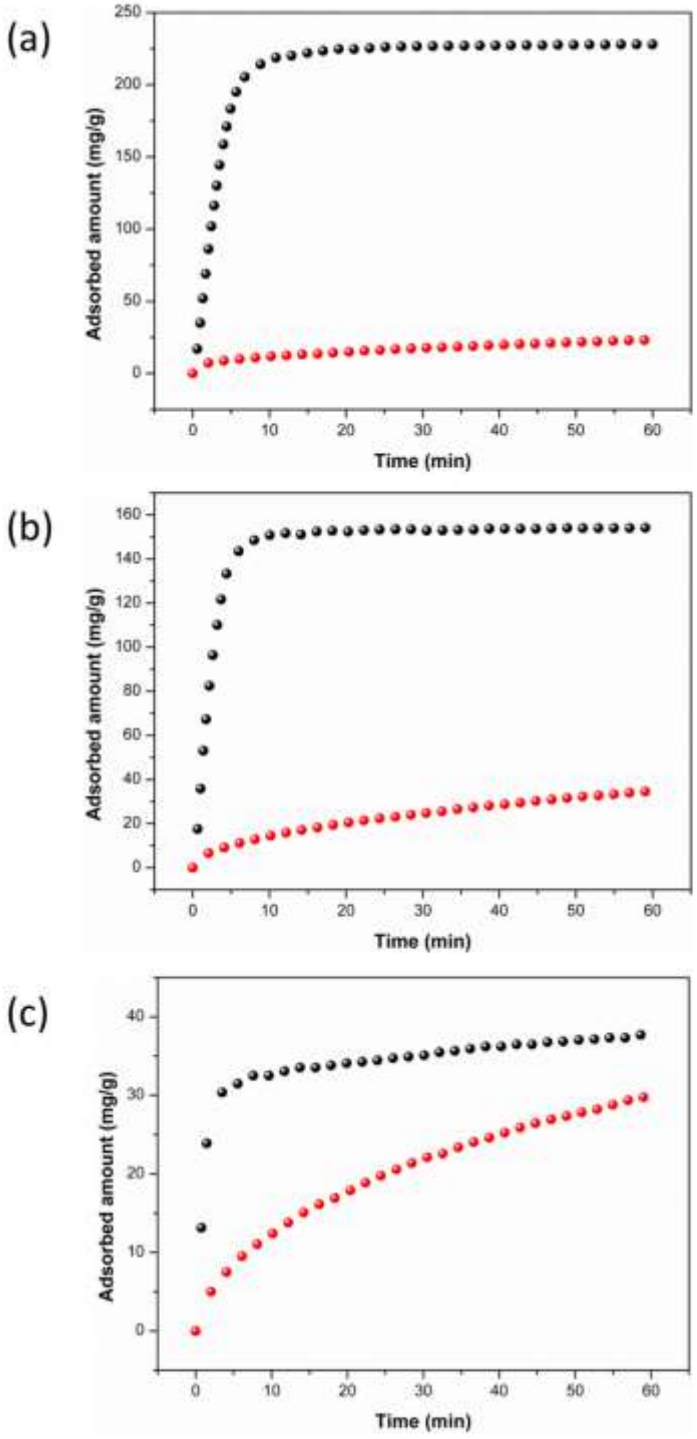

Supplementary Figure 30 │ Adsorption of *n*-hexane (black) and 3-methylpentane (red) on ZIF-8 at (a) 30 °C, (b) 90 °C, and (c) 150°C.

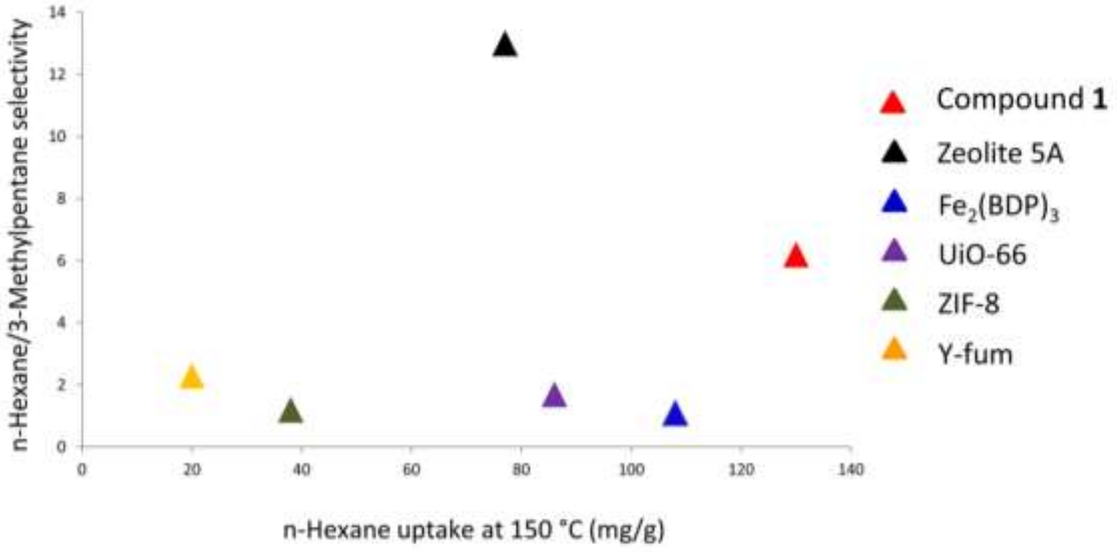

Supplementary Figure 31 | *n*-hexane uptake and *n*-hexane/3-methylpentane uptake ratio for various materials at 150 °C and 100 torr. Selectivity was estimated as the ratio of uptake. Data for $Fe_2(BDP)_3$ were taken from Science 340, 960-964. Data for all other materials were collected in this work.

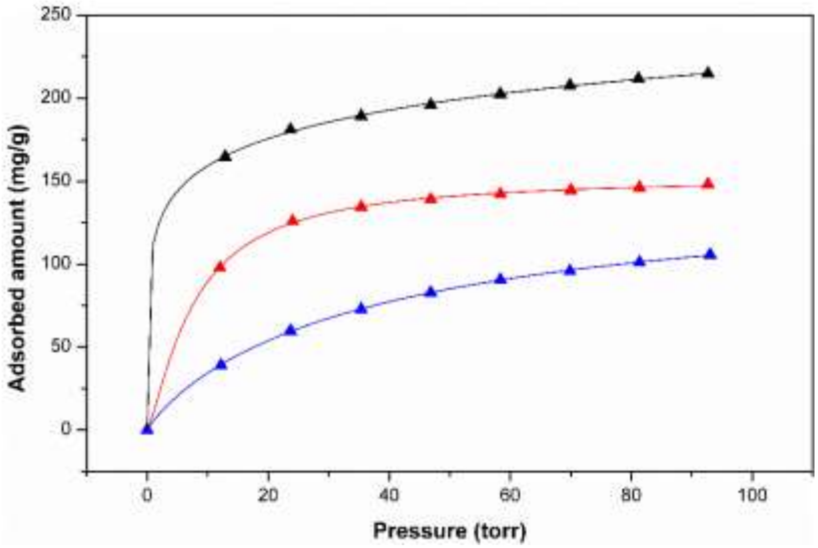

Supplementary Figure 32 │ *n*-hexane adsorption isotherms on compound **2** at 30 °C (black), 90 °C (red) and 150 °C (blue).

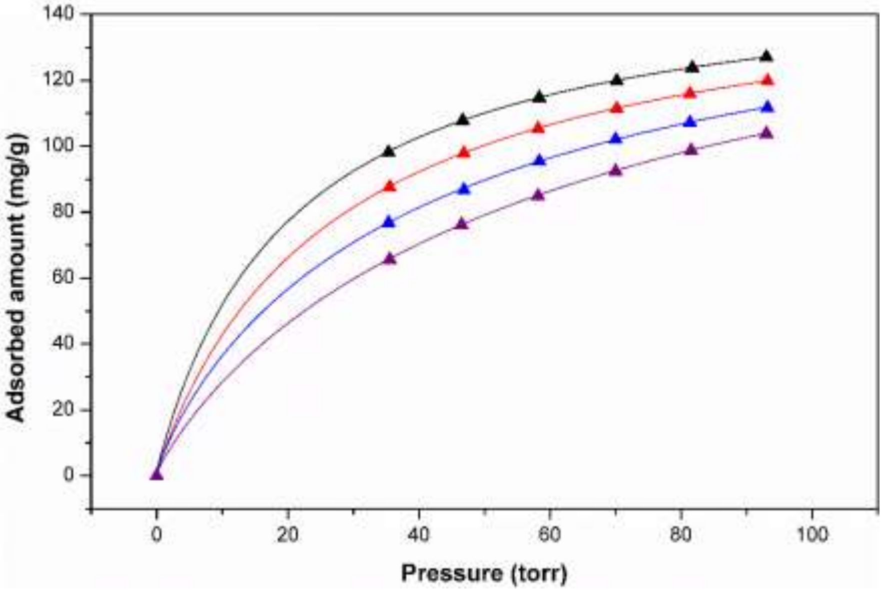

Supplementary Figure 33 │ *n*-hexane adsorption isotherms on compound **2** at 120 °C (black), 130 °C (red), 140 °C (blue), and 150 °C (purple).

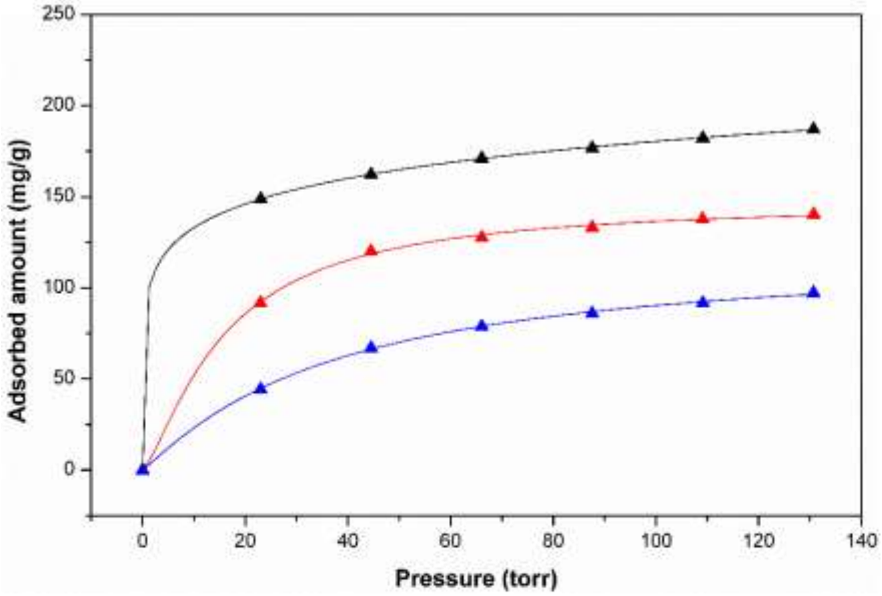

Supplementary Figure 34 | 3-methylpentane adsorption isotherms on compound **2** at 30 °C (black), 90 °C (red) and 150 °C (blue).

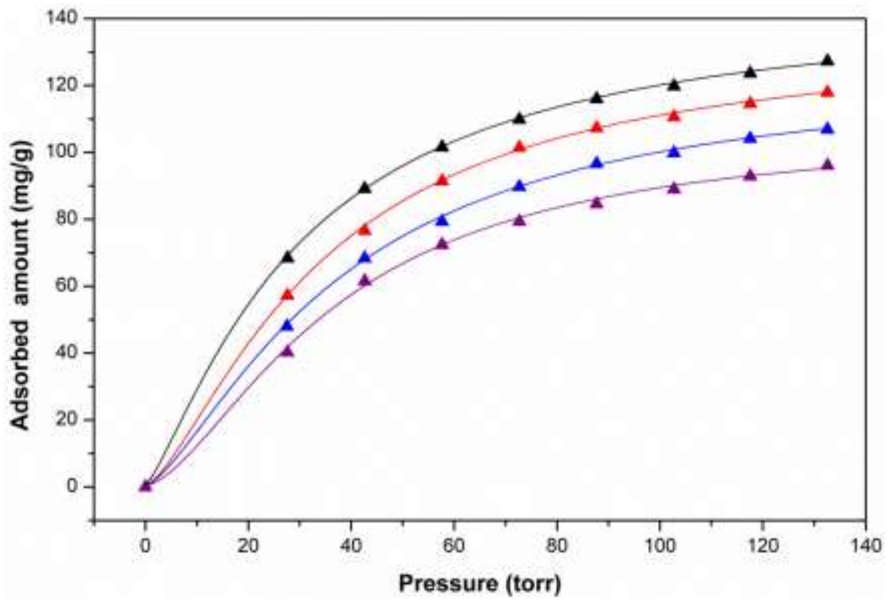

Supplementary Figure 35 | 3-methylpentane adsorption isotherms on compound **2** at 120 °C (black), 130 °C (red) 140 °C (blue) and 150 °C (purple).

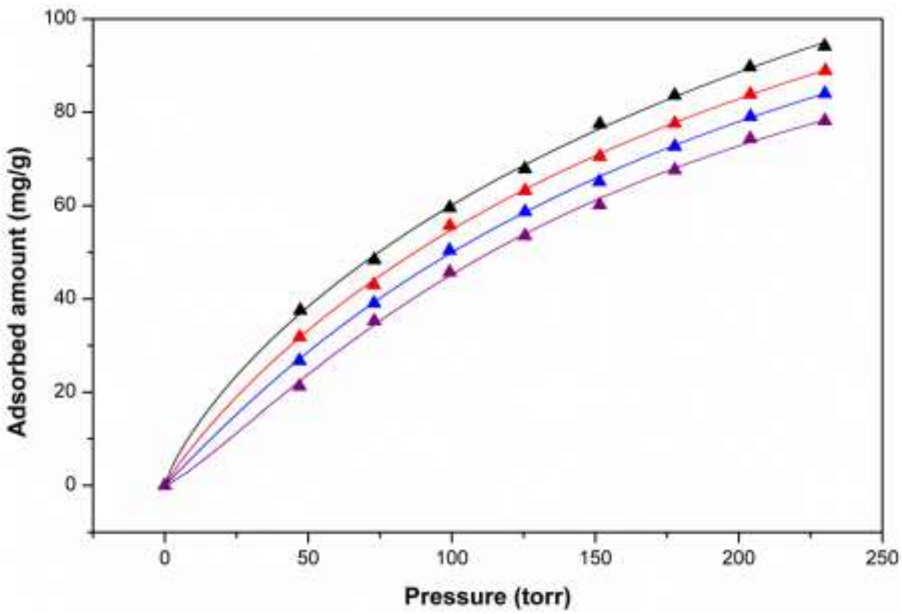

Supplementary Figure 36 │ 2,3-dimethylbutane adsorption isotherms on compound **2** at 120 °C (black), 130 °C (red) 140 °C (blue) and 150 °C (purple).

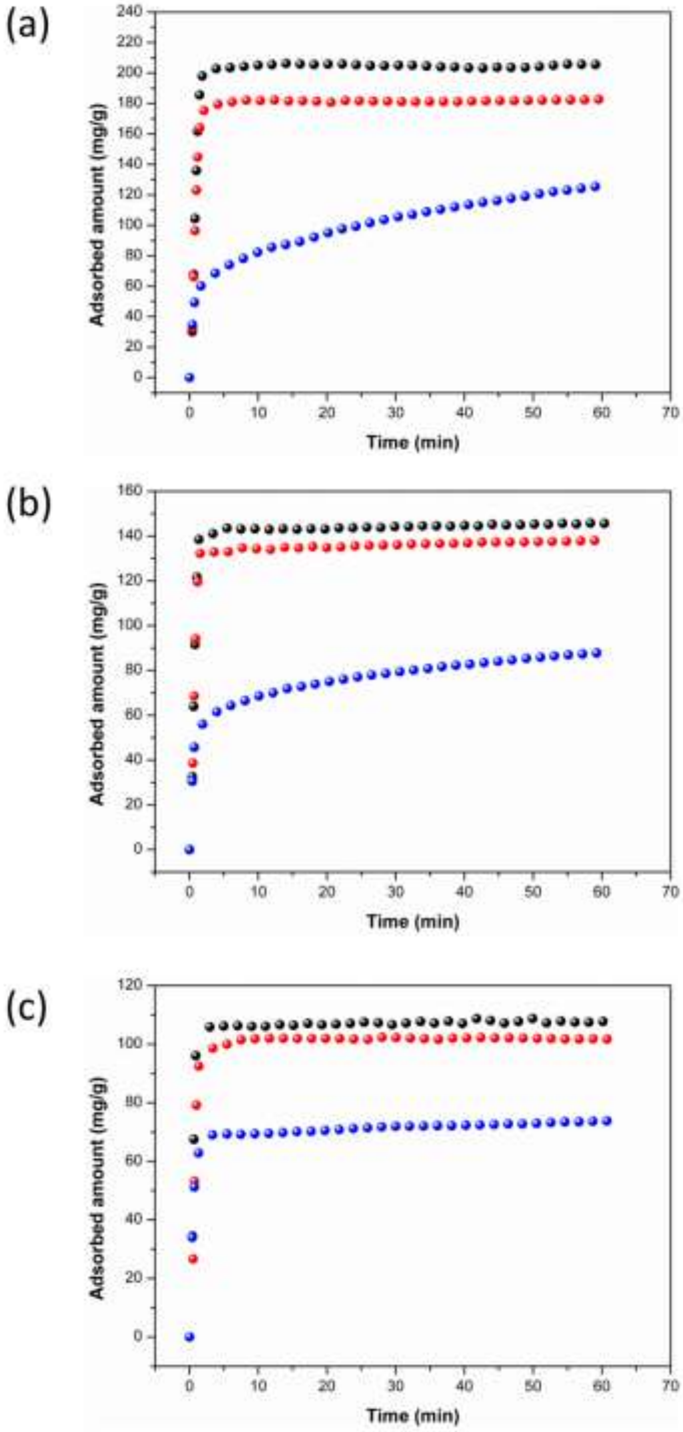

Supplementary Figure 37 | Adsorption of *n*-hexane (black), 3-methylpentane (red) and 2,3-dimethylbutane (blue) on compound **2** at (a) 30 °C, (b) 90 °C, and (c) 150°C.

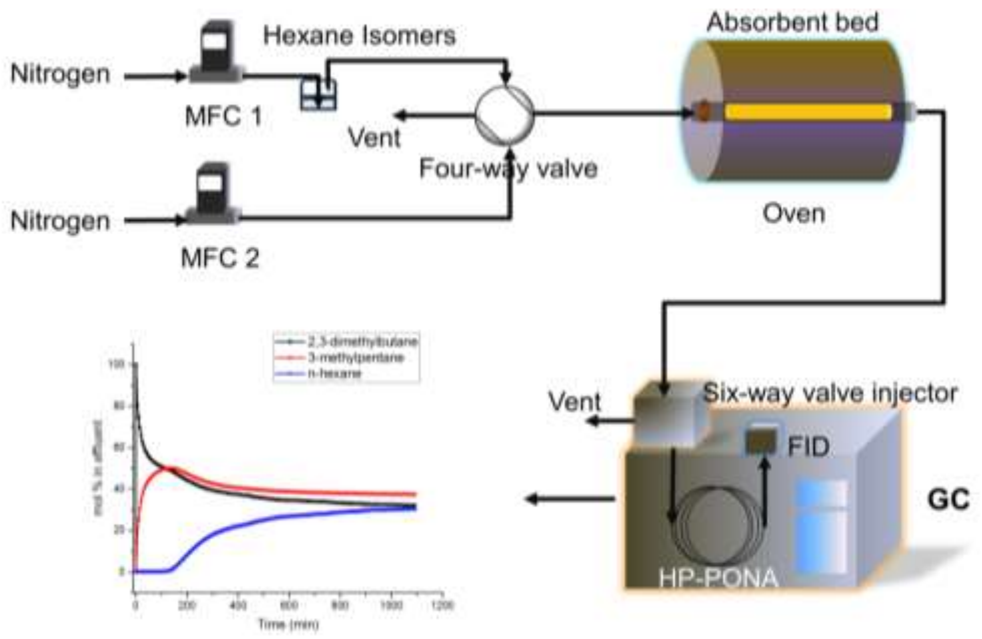

Supplementary Figure 38 │ Schematic representation of column breakthrough experiment. A nitrogen flow (1 cm^3/min) was used to purge the adsorbent. The flow of Nitrogen was then turned off while another dry N_2 at a rate of 1 mL/min bubbled through a mixture of hexane isomers according to the following volumes: 3.50 mL of 2,3-dimethylbutane, 4.22 mL of 3-methylpentane and 5.82 mL of *n*-hexane.[3] The effluent from the column was monitored using an online GC equipped with HP-PONA column and FID.

The absolute adsorbed amount of gas i (q_i) is calculated from the breakthrough curve by the equation:

$$q_i = \frac{F_i \times t_0 - V_{dead} - \int_0^{t_0} F_e \Delta t}{m}$$

where F_i is the influent flow rate of the specific gas (cm^3/min); t_0 is the adsorption time (min); V_{dead} is the dead volume of the system (cm^3); F_e is the effluent flow rate of the specific gas (cm^3/min); and m is the mass of the sorbent (g). The real time RON of the effluent is calculated according to reported literature.[4]

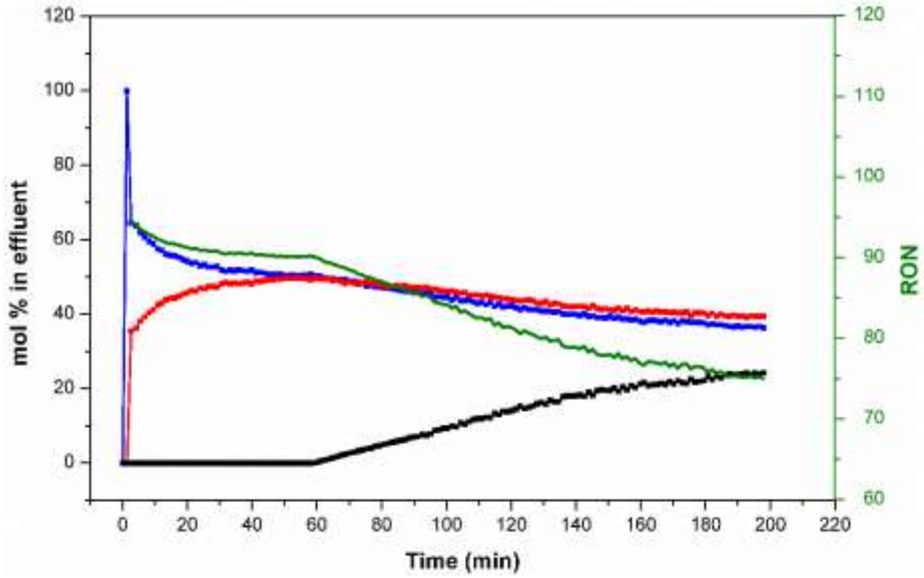

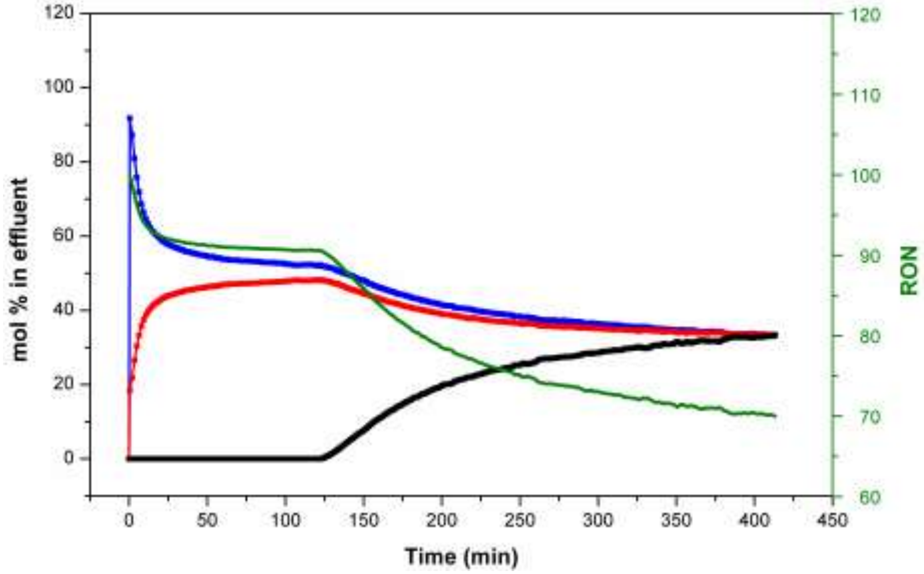

Supplementary Figure 39 │ Separation of *n*-hexane (black), 3-methylpentane (red) and 2,3-dimethylbutane (blue) running through a packed bed of zeolite 5A at 150 °C (top) and 30 °C (bottom). Green curve indicates the RON of the eluted mixture.

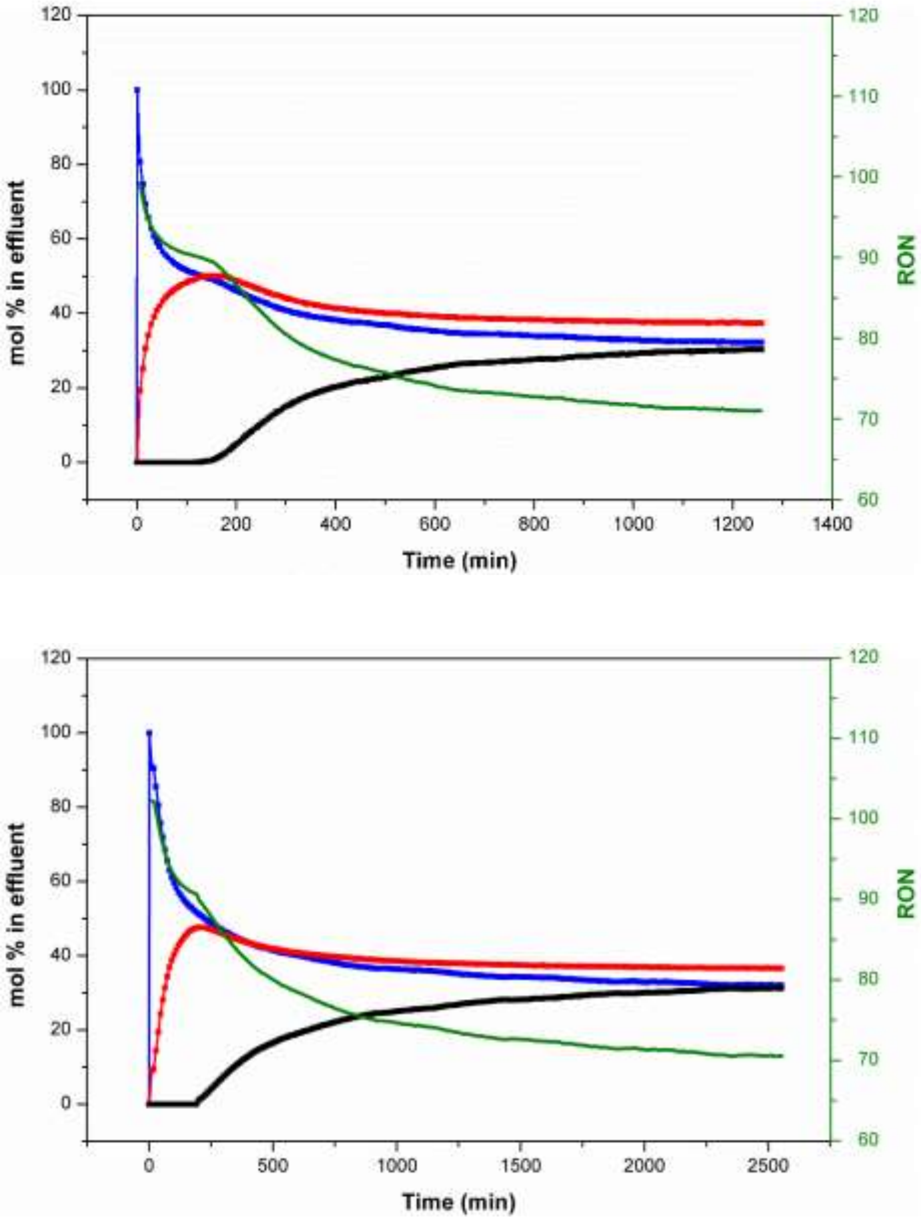

Supplementary Figure 40 | Separation of *n*-hexane (black), 3-methylpentane (red) and 2,3-dimethylbutane (blue) running through a packed bed of compound **1** at 150 °C (top) and 30 °C (bottom). Green curve indicates the RON of the eluted mixture.

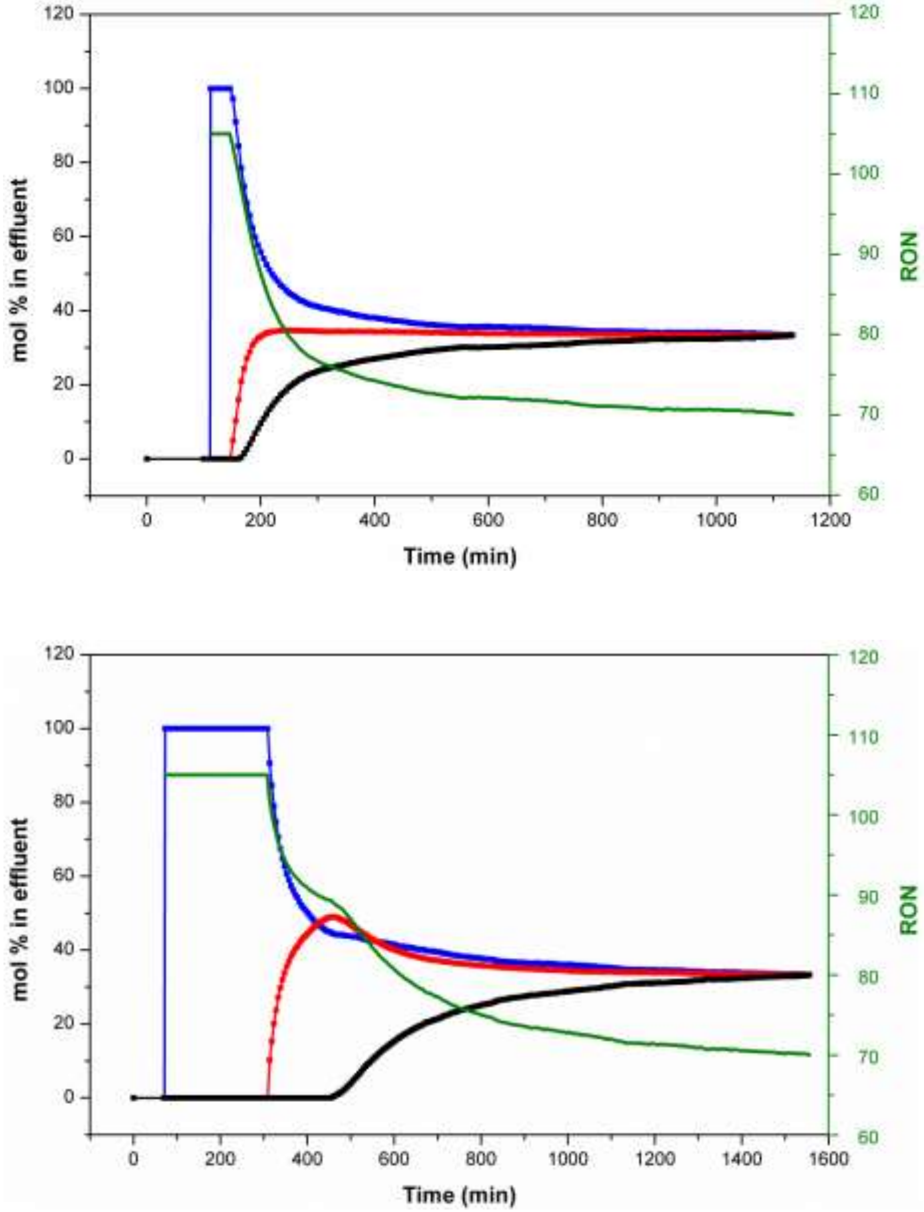

Supplementary Figure 41 │ Separation of *n*-hexane (black), 3-methylpentane (red) and 2,3-dimethylbutane (blue) running through a packed bed of compound **2** at 150 °C (top) and 30 °C (bottom). Green curve indicates the RON of the eluted mixture.

a)

b)

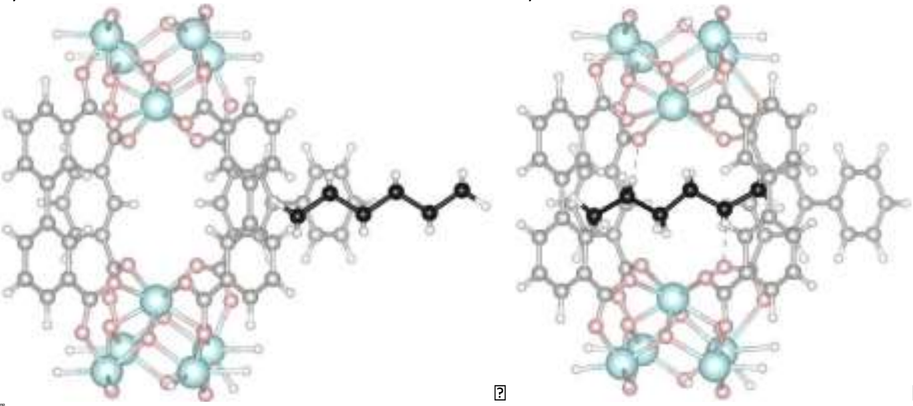

c)

d)

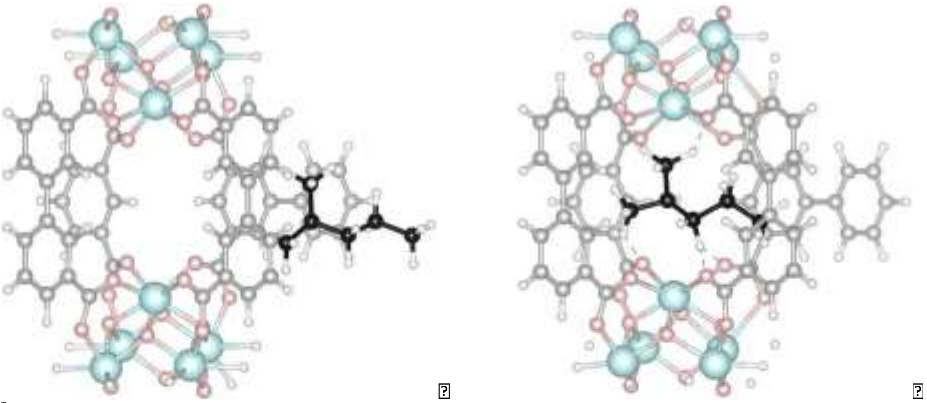

e)

f)

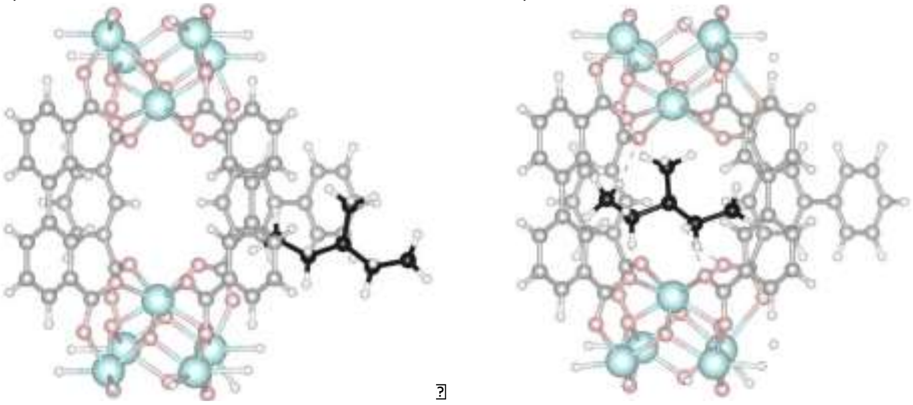

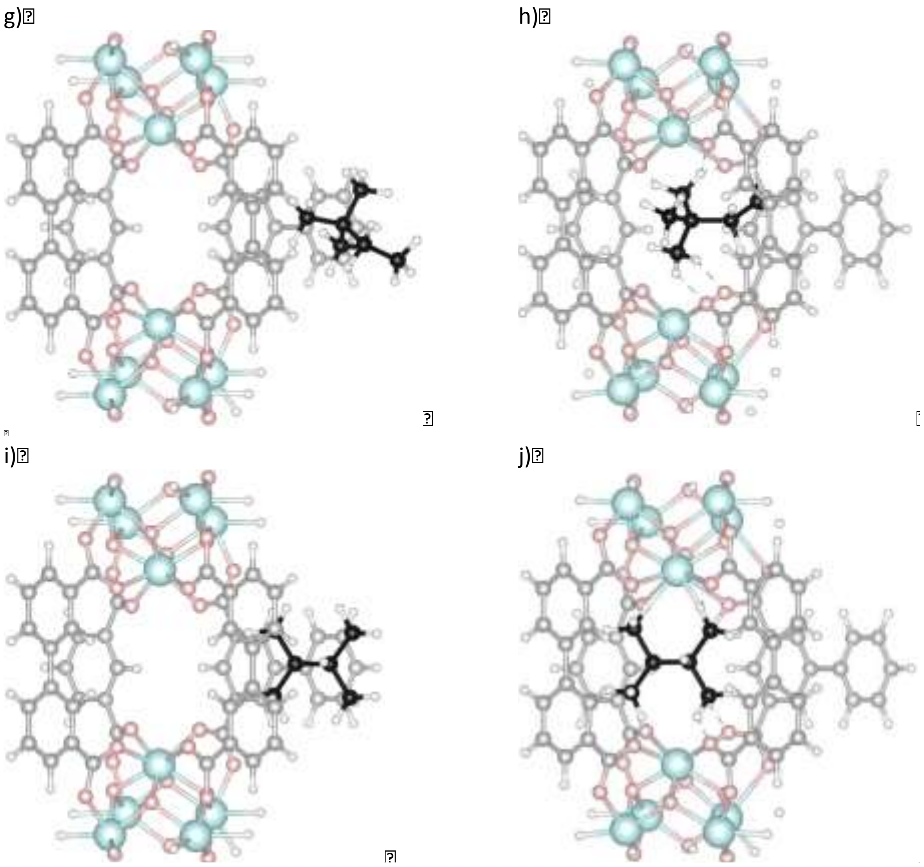

Supplementary Figure 42 │ Models for kinetic energy barrier calculations in compound **1**. Color scheme: C = black, H = white, Zr = cyan, O = red with the MOF pore window in muted colors. (left) Representative structures of the isomers at the entrance of the MOF pore window. The interaction of the isomers with the MOF pore window is negligible in these situations. (right) Representative structures of the isomers inside the MOF pore window where we estimate the kinetic barrier to be the highest, see Supplementary Note 1. (a,b) nHEX, (c, d) 2MP, (e, f) 3MP, (g, h) 22DMB, and (i, j) 23DMB.

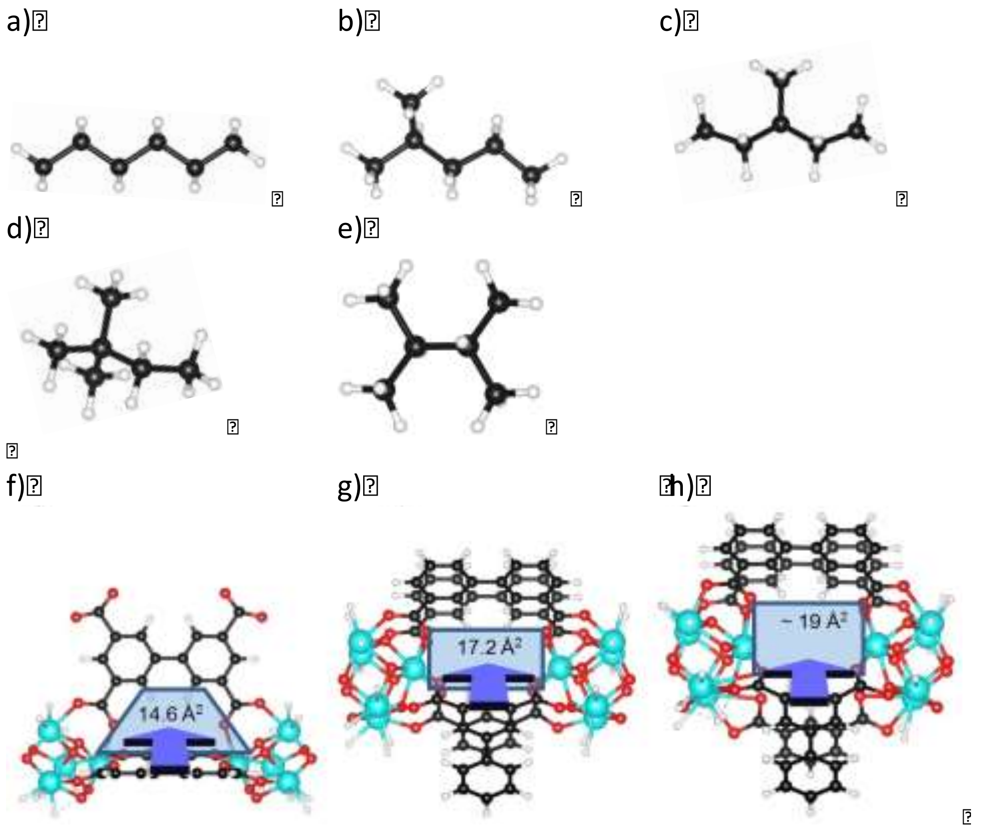

Supplementary Figure 43 │ Simulation Models. Color scheme: C = black, H = white, Zr = cyan, O = red. (a–e) Images of all C6 isomers computationally studied: a) nHEX, b) 2MP, c) 3MP, d) 22DMB, and e) 23DMB. (f–h) The three entrances studied in compound **1**: f) "straight", g) "diagonal", and h) "breathing". In the "straight" model, the guest molecules traverse straight through the pore window opening. In the "diagonal" model, the molecules go along a path that dips down by 45° with respect to the "straight" entrance, which allows the molecules to go through a different opening that is noticeably larger. Finally, the "breathing" model represents the same path as the "diagonal" model, but the opening is temporarily increased in size due to the breathing motion of the MOF. Approximate areas of the various openings are indicated at the center of the figures; in the case of the "breathing" model, the indicated area is a typical value during the breathing motion.

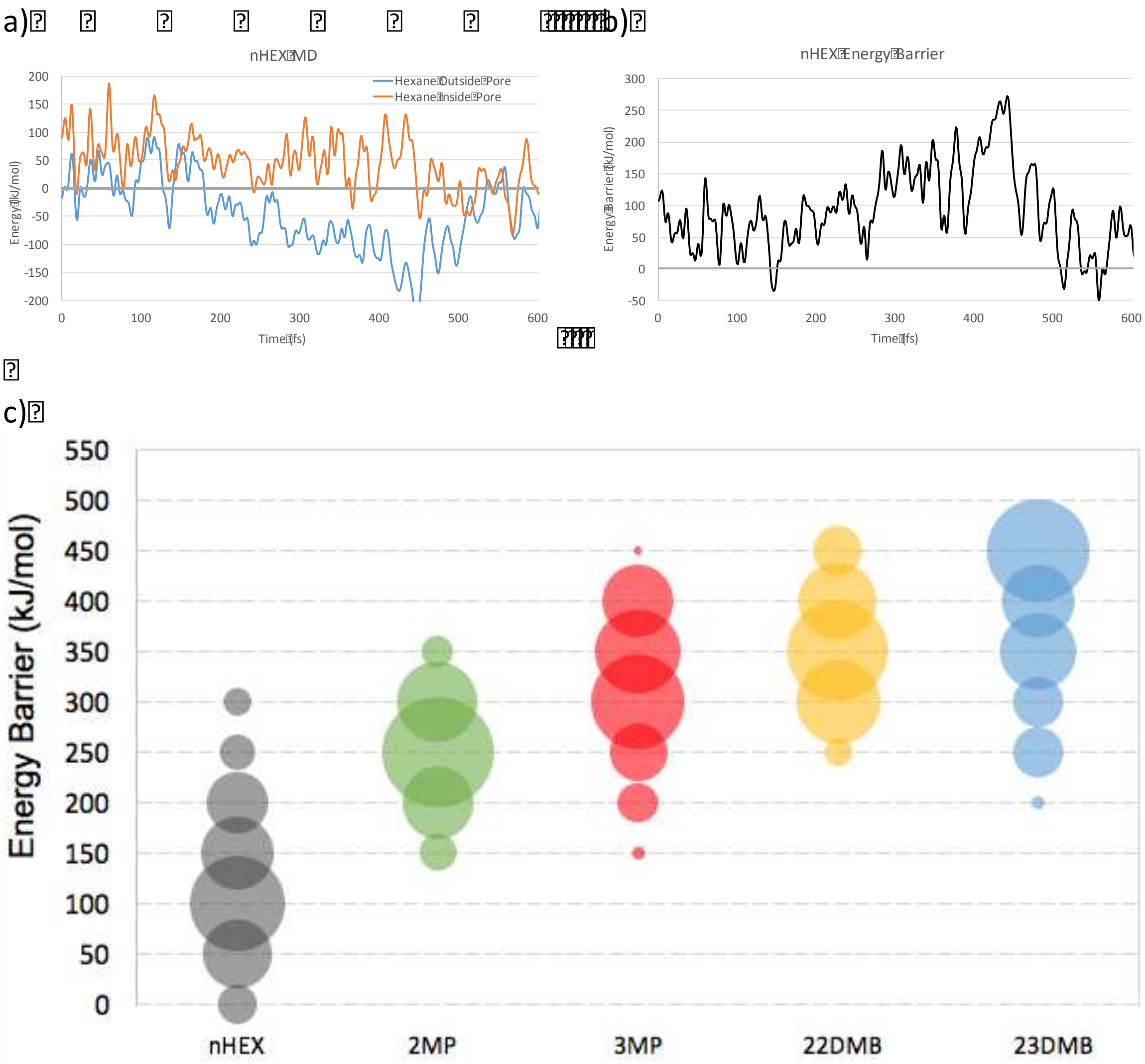

Supplementary Figure 44 | Time and temperature dependent energy barriers for compound **1**. (a,b) Total energy fluctuations for nHEX at 150 °C along with the calculated AIMD energy barrier. The blue line indicates the total energies of the system when the isomer is outside the MOF pore, the orange line is the total energy with the isomer inside the MOF pore (see Supplementary Fig. 42), and the energy barrier (defined as the difference between the orange and blue line) is depicted as black in the right panel. The nHEX AIMD results are used as a representative here and results for 2MP, 3MP, 22DMB, and 23DMB look qualitatively similar. Note that the breathing mode of the MOF pore is observable in these AIMD results as a slow

increase and decrease in energy with a period much larger than the fine fluctuations of the AIMD time steps; from these data we estimate the breathing mode at less than 100 cm^{-1}. (c) Summary of the AIMD results for all isomers in form of a bubble plot, where the AIMD trajectories (a and b) are analyzed up to the point where the isomer leaves the pore (see Supplementary Note 1). The size of each circle corresponds to the amount of time spent in the increment. A larger circle thus indicates more time steps with a barrier in that increment range.

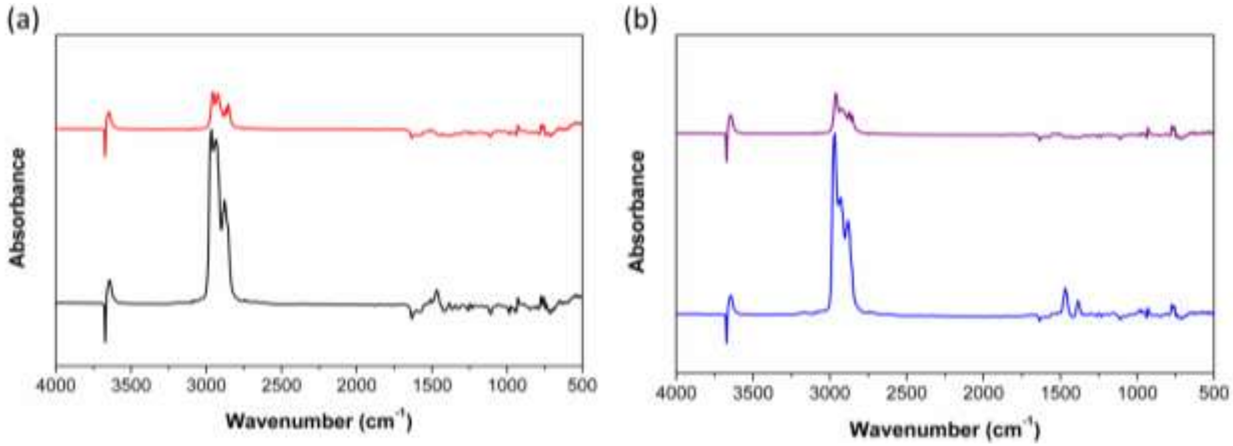

Supplementary Figure 45 | IR spectra of (a) nHEX and (b) 3MP adsorbed compound **2** (bottom spectra) and after 1 min evacuation (top spectra). All spectra are normalized by the reference spectra recorded prior to adsorption.

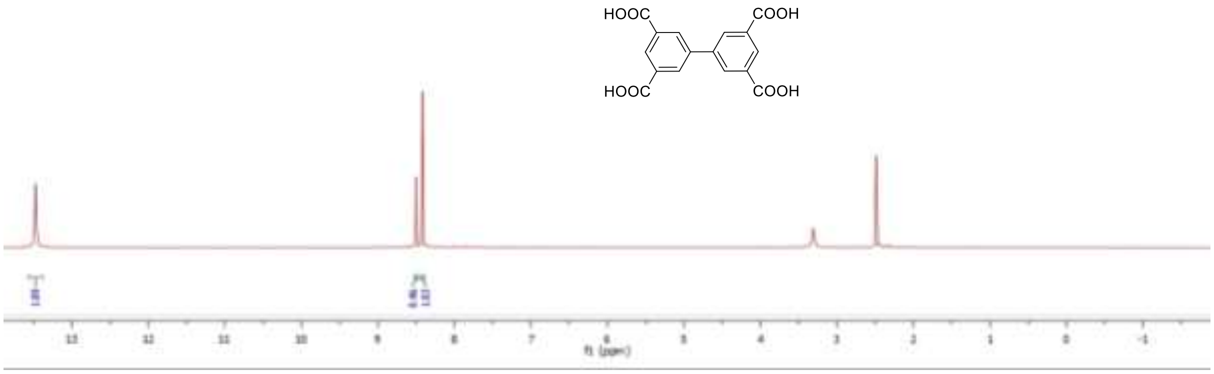

Supplementary Figure 46 │ ^{1}H NMR spectrum of H_4bptc (DMSO- d^6).

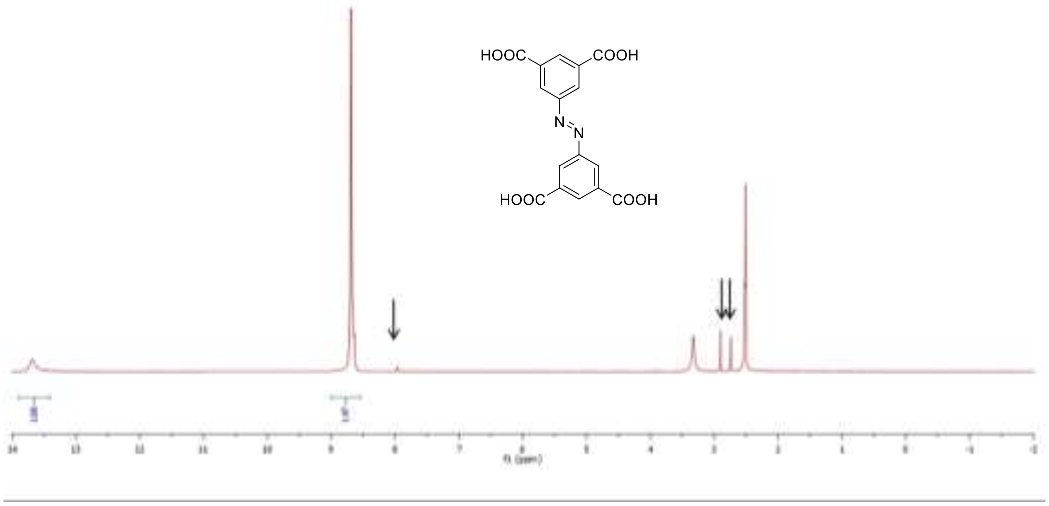

Supplementary Figure 47 │ ^{1}H NMR spectrum of H_4abtc (DMSO-d^6). (Peaks marked by black arrows are associated to DMF residual).

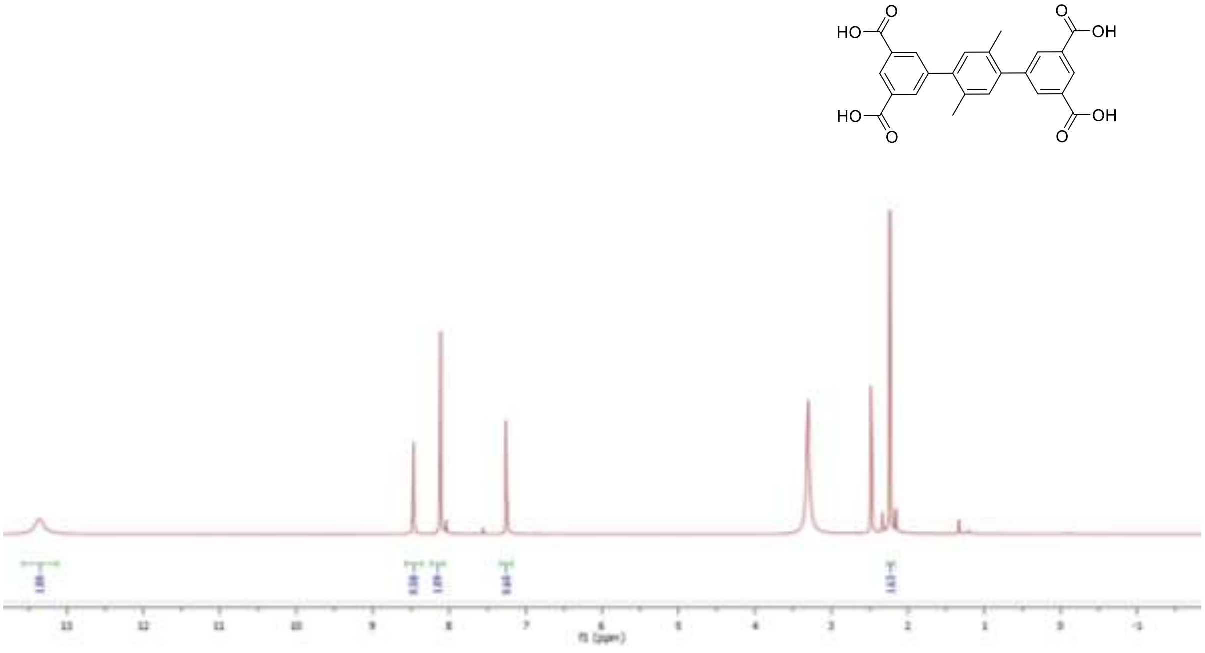

Supplementary Figure 48 │ ^{1}H NMR spectrum of H_4bptc-$(Me)_2$ (DMSO- d^6).

Supplementary Table 1 │ Crystal data and structure refinement of compound **1**.

Formula	$Zr_6O_4(OH)_4(bptc)_3$
Wavelength	1.5406 Å Cu-Kα_1
Crystal system	Cubic
Space group	*Im*-3
Unit cell dimensions	a = 24.3597 (3) Å
Volume	14455.0 (5) Å^3
Z	8
2Theta range for data refinement	5°< 2θ < 70°
Number of parameters	30
Number of data points	5000
Number of restrains	6 for Zr-O bond distances, 10 for O-Zr-O bond angles, 4 for C-O bond distances, 2 for C-C bond distances
Refinement method	Rietveld refinement
$R_p/R_{wp}/R_{exp}$	0.0391/0.0586/0.0273
R_B	0.0381

Supplementary Table 2 │ Crystal data and structure refinement for compound **2**.

Empirical formula	C32 H20 N4 O32 Zr6	
Formula weight	1519.84	
Temperature	100(2) K	
Wavelength	0.7749 Å	
Crystal system	Monoclinic	
Space group	C2/m	
Unit cell dimensions	a = 25.4692(11) Å	α = 90°.
	b = 36.3589(15) Å	β = 122.260(2)°.
	c = 21.5275(9) Å	γ = 90°.
Volume	16857.8(13) $Å^3$	
Z	8	
Density (calculated)	1.198 Mg/m^3	
Absorption coefficient	0.965 mm^{-1}	
F(000)	5888	
Crystal size	0.130 x 0.020 x 0.020 mm^3	
Theta range for data collection	2.397 to 27.868°.	
Index ranges	-30<=h<=25, 0<=k<=43, 0<=l<=25	
Reflections collected	94479	
Independent reflections	22502 [R(int) = 0.1156]	
Completeness to theta = 27.706°	98.6 %	
Absorption correction	Semi-empirical from equivalents	
Max. and min. transmission	0.981 and 0.612	
Refinement method	Full-matrix least-squares on F^2	
Data / restraints / parameters	22502 / 1202 / 678	
Goodness-of-fit on F^2	1.027	
Final R indices [I>2sigma(I)]	R1 = 0.0848, wR2 = 0.2296	
R indices (all data)	R1 = 0.1311, wR2 = 0.2742	
Extinction coefficient	n/a	
Largest diff. peak and hole	2.918 and -1.470 e.$Å^{-3}$	

Supplementary Table 3 │ Crystal data and structure refinement for compound **3**.

Empirical formula	C14 H17 O16 Zr3	
Formula weight	714.93	
Temperature	100(2) K	
Wavelength	0.7749 Å	
Crystal system	Orthorhombic	
Space group	Imma	
Unit cell dimensions	a = 25.2379(14) Å	α = 90°.
	b = 27.7529(16) Å	β = 90°.
	c = 15.2344(10) Å	γ = 90°.
Volume	10670.6(11) $Å^3$	
Z	8	
Density (calculated)	0.890 Mg/m^3	
Absorption coefficient	0.757 mm^{-1}	
F(000)	2792	
Crystal size	0.200 x 0.070 x 0.040 mm^3	
Theta range for data collection	2.337 to 22.990°.	
Index ranges	-25<=h<=25, -27<=k<=27, -15<=l<=15	
Reflections collected	21998	
Independent reflections	2981 [R(int) = 0.0642]	
Completeness to theta = 22.990°	98.2 %	
Refinement method	Full-matrix least-squares on F^2	
Data / restraints / parameters	2978 / 275 / 172	
Goodness-of-fit on F^2	3.131	
Final R indices [I>2sigma(I)]	R1 = 0.1484, wR2 = 0.3954	
R indices (all data)	R1 = 0.1896, wR2 = 0.4420	
Extinction coefficient	n/a	
Largest diff. peak and hole	4.545 and -1.103 e.$Å^{-3}$	

Supplementary Table 4 | Summary of Zr-MOFs built on tetratopic linkers.

MOF	Space group	Topology	Coordination of nodes	Shape of ligand	Ligand L/W (Å)	Coordination figure of Zr_6 cluster	Ref.
					Aspect ratio		
Zr-bptc	Im-3	**ftw**	4,12-c	rectangle	7.40/5.09<br>1.45	cubooctahedron	This work
Zr-abtc	C2/m	**scu**	4,8-c	rectangle	8.95/5.03<br>1.78	tetragonal prism	This work
Zr-tptc-$(Me)_2$	Imma	**lvt**	4,4-c	rectangle	11.27/4.94<br>2.28	square	This work
1-X	Pm-3	**ftw**	4,12-c	rectangle	11.19/9.69<br>1.15	cubooctahedron	[5]
NU-1100	Im-3	**ftw**	4,12-c	rectangle	16.01/15.44<br>1.03	cubooctahedron	[6]
Zr-PTBA	R-3r	**ftw**	4,12-c	rectangle	15.71/15.69<br>1.00	cubooctahedron	[7]
Zr-BTBA	R-3r	**ftw**	4,12-c	square	15.76/15.76<br>1.00	cubooctahedron	[7]
PCN-230	Pm-3m	**ftw**	4,12-c	square	32.27/32.27<br>1.00	cubooctahedron	[8]
NU-1105	Pm-3m	**ftw**	4,12-c	square	23.82/23.82<br>1.00	cubooctahedron	[9]
NU-1104	Pm-3m	**ftw**	4,12-c	square	22.63/22.63<br>1.00	cubooctahedron	[10]
CPM-99(Fe)	Pm-3m	**ftw**	4,12-c	square	19.13/19.13<br>1.00	cubooctahedron	[11]
FJI-H6 (NU-1102)	Pm-3m	**ftw**	4,12-c	square	19.12/19.12<br>1.00	cubooctahedron	[12]
CPM-99 (NU-1102)	Pm-3m	**ftw**	4,12-c	square	19.10/19.10<br>1.00	cubooctahedron	[11]
CPM-99 (Zn)	Pm-3m	**ftw**	4,12-c	square	19.10/19.10<br>1.00	cubooctahedron	[11]
FJI-H6 (Cu)	Pm-3m	**ftw**	4,12-c	square	19.10/19.10<br>1.00	cubooctahedron	[12]
CPM-99 (Co)	Pm-3m	**ftw**	4,12-c	square	19.06/19.06<br>1.00	cubooctahedron	[11]
NU-1102	Pm-3m	**ftw**	4,12-c	square	19.04/19.04<br>1.00	cubooctahedron	[10]
PCN-228’	Pm-3m	**ftw**	4,12-c	square	19.03/19.03<br>1.00	cubooctahedron	[8]
PCN-94	Pm-3m	**ftw**	4,12-c	square	14.73/14.73<br>1.00	cubooctahedron	[13]
MOF-525	Pm-3m	**ftw**	4,12-c	square	13.30/13.30<br>1.00	cubooctahedron	[14]
MOF-535	Pm-3m	**ftw**	4,12-c	square	13.26/13.26<br>1.00	cubooctahedron	[14]
PCN-128Y	P6/mm	**csq**	4,8-c	rectangle	17.75/10.90<br>1.63	Tetragonal prism	[15]
PCN-128W	P6/mm	**csq**	4,8-c	rectangle	17.26/11.89<br>1.45	Tetragonal prism	[15]
UCMC-313	P6/mm	**csq**	4,8-c	rectangle	14.99/11.27<br>1.33	Tetragonal prism	[16]
MOF-545-Fe	P6/mm	**csq**	4,8-c	rectangle	13.68/12.23<br>1.12	Tetragonal prism	[14]
PCN-222	P6/mm	**csq**	4,8-c	rectangle	13.36/12.64<br>1.06	Tetragonal prism	[17]

NU-1000	P6/mm	**csq**	4,8-c	rectangle	12.08/12.04<br>1.00	Tetragonal prism	[18]
BUT-15	I41/amd	**sqc**	4,8-c	rectangle	16.14/11.97<br>1.35	Tetragonal prism	[19]
BUT-14	I41/amd	**sqc**	4,8-c	rectangle	16.14/12.06<br>1.34	Tetragonal prism	[19]
PCN-225	I41/amd	**sqc**	4,8-c	rectangle	13.77/12.02<br>1.15	Tetragonal prism	[20]
NUPF-1	Cmcm	**scu**	4,8-c	rectangle	24.07/20.13<br>1.20	Tetragonal prism	[21]
PCN-223(Fe)	P6/m	**shp**	4,12-c	rectangle	13.59/12.34<br>1.10	Hexagonal prism	[22]
PCN-224 (Co)	Im-3m	**she**	4,6-c	square	13.04/13.04<br>1.00	Hexagon	[23]
PCN-224(Fe)	Im-3m	**she**	4,6-c	square	13.03/13.03<br>1.00	Hexagon	[24]
PCN-224(Co)	Im-3m	**she**	4,6-c	square	13.02/13.02<br>1.00	Octahedron	[24]
Ir-PMOF-1(Zr)	Im-3m	**she**	4,6-c	square	13.00/13.00<br>1.00	Hexagon	[25]
PCN-224(Ni)	Im-3m	**she**	4,6-c	square	12.97/12.97<br>1.00	Hexagon	[26]
PCN-224(Fe)	Im-3m	**she**	4,6-c	square	12.95/12.95<br>1.00	Hexagon	[24]
PCN-224(Zr)	Im-3m	**she**	4,6-c	square	12.86/12.86<br>1.00	Hexagon	[26]
MOF-841	I4/m	**flu**	4,8-c	tetrahedron		Tetragonal prism	[27]
PCN-521	I4/m	**flu**	4,8-c	tetrahedron		Tetragonal prism	[28]
UCMC-312	Fmmm	**flu**	4,8-c	tetrahedron		Tetragonal prism	[16]
Zr-TCPS	I4/m	**flu**	4,8-c	tetrahedron		Tetragonal prism	[29]
MOF-812	C2/c	**ith**	4,12-c	tetrahedron		cubooctahedron	[27]

Supplementary Table 5 | Topology summary of reported MOFs on polytopic ligands (transitivity given in square brackets, number of reported structures given in parentheses)

	Polytopic ligand (56)				
CN of Zr_6O_8	4 (rectangular, square)	4 (tetrahedral)	3	2 and 3	3 and 4
12	[21] **ftw** (18 + *this work*)<br>[21] **shp** (TUTWUC)	**ith** [21] (BOHWOM)	**llj** [22] (OWILAJ)		
10					3,4,10-new OQIKUW
8	all[21] **csq** (6), **sqc** (3), **scu** (QAGBUY, *this work*)	**flu** [21] (4)	**the** [21] (3)	**tfz-d** [22] (OQIKOQ)	
6	**she** [21] (7)		**spn** [21] (3)<br>2D [21] **kgd** (3)		
4 square	**lvt** [11] *(this work)*				

**Supplementary Table 6 | ** Topology summary of reported MOFs on ditopic ligands (transitivity given in square brackets, number of reported structures given in parentheses)

CN of Zr_6O_8	Ditopic ligand (145)
12	**fcu** [11] (95 single + 7 2fold), **bcu-x-**12-P42/mmc [13] (4)
11	**ela** [13] (3)
10	**bct** [12] (11), **fcu-**10-P42/mmc [12] (2)
8	**bcu** [11] (14), **reo** [11] (4 bent ligand), **hex** [12](1), **bon** [24] (1)
4	2D **sql** [11] (7)

Supplementary Table 7 │ Porosity characterization before and after water treatment (Water treatment: ~100 mg of sample immersed in ~20 mL of water which is heated at 80 °C for 24 hours).

	BET surface area (m^2/g)	
	Before water treatment	After water treatment
Compound **1**	1030	1035
Compound **2**	1318	1250
UiO-67	2299	473

Supplementary Table 8 │ Calculated kinetic energy barriers for nHEX in compound **1**. 0 K temperature results are obtained with ground-state *ab initio* calculations; the finite temperature results are found via AIMD simulations (see Supplementary Note 1).

Model	Temp.	Energy Barrier (kJ/mol)
Straight	0 K	399
Diagonal	0 K	358
Breathing	0 K	174
Breathing	150 (°C)	0 – 187

Supplementary Table 9 │ Energy barrier statistics for compound **1** at 150 °C. Average energy barriers and percent time spent below a barrier of 100 kJ/mol.

Model	Time Averaged Energy Barrier (kJ/mol)	% Time with Energy Barrier < 100 kJ/mol
nHEX	92	59%
2MP	217	0%
3MP	284	0%
22DMB	316	0%
23DMB	406	0%

Supplementary Note 1 │ Computational details

For completeness, all C6 alkane isomers were studied with *ab initio* methods for adsorption into compound **1**. In particular, energy barriers for entrance of nHEX, 2MP, 3MP, 22DMB, and 23DMB were calculated. Due to the large size of the unit cell of compound **1** (~850 atoms), cutouts were taken directly from the experimental CIF file to represent the windows the isomers pass through (Supplementary Fig. 42), consisting of 136 atoms; the individual periodic images of the cutout clusters were separated by at least 10 Å to minimize spurious interactions. Each cutout was properly hydrogen terminated and optimized at the density functional theory (DFT) level with VASP[30,31] before the energy barriers were calculated. The PBE exchange-correlation functional was used and PAW pseudopotentials were implemented with an energy cutoff of 600 eV; only the Γ point was used.

As explained in the main text, at the beginning of our investigation various mechanisms for entrance were tested: ("straight", "diagonal", and "breathing", see Supplementary Fig. 43) for nHEX. The corresponding results in Supplementary Table 8 suggest that a full treatment of temperature effects via AIMD simulations is necessary. Diffusion events over large barriers are statistically rare events on the timescale accessible through AIMD calculations, preventing a direct assessment of the diffusion barrier. We thus estimate the diffusion barrier as the difference in total energy of the isomer inside the MOF pore window and the isomer at the entrance (just outside the MOF pore window), as depicted in Supplementary Fig. 42; both AIMD runs are performed independently of each other. For the calculations where the isomer is inside the MOF pore window, we place the isomer at the point that we estimate to have the highest barrier. We found those points through an initial ground-state (corresponding to 0 K) transition-state search algorithm[32,33] and argue that the point of highest barrier at finite temperatures does not deviate much from the 0 K

points. All such found states within the pore windows are depicted in Supplementary Fig. 42. As a representative, the total energies at each time step for a 600 fs trajectory for nHEX are plotted in Supplementary Fig. 44 alongside the time-dependent energy barrier, but we also obtained qualitatively similar data for 2MP, 3MP, 22DMB, and 23DMB. An energy barrier of 100 kJ/mol (~1 eV) is approximately an upper limit to have non-negligible statistical probabilities for molecules to pass through the pore window at the studied of 150 °C, and the percent of time spent below a 100 kJ/mol barrier is presented in Supplementary Table 9. Only the nHEX energy barriers cross below the 100 kJ/mol value, while the branched isomers do not even come close. AIMD calculations were run for over 1.5 ps and data was collected after a 200 fs thermalization period. Note that the molecules inside the pore window represent metastable states and they all come out of the pore eventually during the course of the AIMD run, typically towards the end of our 1.5 ps runs. However, 23DMB is particularly metastable, as can be inferred by its large barrier in Supplementary Fig. 44 and Supplementary Table 9, such that this isomer leaves the pore around 600–700 fs.

Supplementary Note 2 │ Notes on refinement of compound **2**.

Several crystals were tried and this was the best crystal. The diffraction pattern showed twinning. Using Cell_now three orientation matrices were determined, the relationship between these components was determined to be 90 degrees about real axis 0 1 1 and 180 degrees about reciprocal axis 1 0 0. The data were integrated using the three matrices in SAINT, TWINABS was used to produce a merged HKLF4 file, for structure solution and initial refinement, and HKLF5 file for final structure refinement. The HKLF5 file contained the merged reflections first component and those that overlapped with this component, which were split into 3 reflections. TWINABS indicated the twin faction to be 39:39:22. The structure was solved using the HKLF4 file, but the best refinement was given by the HKLF5 file.

All fully occupied non-hydrogen atoms were refined anisotropically. Hydrogen atoms were placed geometrically on the carbon atoms constrained and refined using a riding model. Hydrogen atoms could neither be found nor placed on the water molecules and μ-3 oxygen atoms and therefore they were omitted from the refinement but not the chemical formula. Displacement and geometrical restraints were necessary as the quality of the data was not ideal due to the twinning.

Refined as a 3-component twin.

;

_vrf_PLAT602_Compound **2**

;

PROBLEM: VERY LARGE Solvent Accessible VOID(S) in Structure ! Info

RESPONSE: There is a large solvent channel in the structure but as a result of the twinning no clear solvent molecules could be found.

;

_vrf_PLAT213_ Compound **2**

;

PROBLEM: Atom C32 has ADP max/min Ratio ..... 4.9 oblate

RESPONSE: The twinning has had an adverse effect of the data which require significant displacement parameter restraints. Even tighter restraints would be required, so it was decided to leave it as it is.

;

_vrf_PLAT342_ Compound **2**

;

PROBLEM: Low Bond Precision on C-C Bonds .... 0.03531 Ang.

RESPONSE: The twinning has had an adverse effect of the data.

;

_vrf_PLAT430_ Compound **2**

;

PROBLEM: Short Inter D...A Contact O9 .. O26 .. 2.70 Ang.

RESPONSE: Ligand carboxylate groups are in close approximate to mu3 hydroxides in the cluster or neighboring carboxylate groups.

;

_vrf_PLAT910_ Compound **2**

;

PROBLEM: Missing # of FCF Reflection(s) Below Theta(Min). 13 Note

RESPONSE: The unit cell is reasonable large and these low angle reflections are probably missing due to the beam stop.

Supplementary Note 3 │ Notes on refinement of compound **3**.

Numerous crystals were tried but all show strong diffuse scattering in the form of diffuse Bragg peaks and scattering between them. The data were cut at 1.0 Angstroms above the I/sigma(I) < 2 meaning on average the data was not observed. The above indicated a high degree of disorder in the system. All non-hydrogen atoms were refined anisotropically. Hydrogen atoms were placed geometrically, then constrained and refined using a riding model. It was not possible to find or place the methyl hydrogen atoms therefore they were omitted from the refinement but not the chemical formula. Geometrical and displacement parameter restraints were used to model the structure. SQUEEZE was used to remove the electron density in the porous that could not be modelled as solvent. SQUEEZE reported Solvent Accessible Volume of 7192 $Å^3$ and Electrons Found in S.A.V. to be 1112. As there is a mixture of DMF and water used it was not possible to approximate the amount of each therefore no solvent was included in the chemical formula. Base on the SQUEEZE output there could be anywhere from around 5 molecules for DMF to around 100 of water molecules.

_vrf_THETM01_ Compound **3**

PROBLEM: The value of sine(theta_max)/wavelength is less than 0.550
RESPONSE: The data were cut at 1.0 Angstroms above above the I/sigma(I) < 2 meaning on average the data was not observed.

_vrf_PLAT973_ Compound **3**

PROBLEM: Check Calcd Positive Residual Density on Zr1 4.97 eA-3
RESPONSE: The crystals all showed strong diffuse scattering in the form of diffuse Bragg peaks and scattering between them. As the model does not account for this type of disorder this is believe to be the source of the high electron density on the Zr.

_vrf_PLAT084_ Compound **3**

PROBLEM: High wR2 Value (i.e. > 0.25) .... 0.44 Report

RESPONSE: The crystals all showed strong diffuse scattering in the form of diffuse Bragg peaks and scattering between them. As the model does not account for this type of disorder this is results in a high wR2.

_vrf_PLAT094_ Compound **3**

PROBLEM: Ratio of Maximum / Minimum Residual Density .... 4.12 Report
RESPONSE: The crystals all showed strong diffuse scattering in the form of diffuse Bragg peaks and scattering between them. As the model does not account for this type of disorder

_vrf_PLAT097_ Compound **3**

PROBLEM: Large Reported Max. (Positive) Residual Density 4.55 eA-3
RESPONSE: The crystals all showed strong diffuse scattering in the form of diffuse Bragg peaks and scattering between them. As the model does not account for this type of disorder this is believe to be the source of the high electron density on the Zr.

_vrf_PLAT342_ Compound **3**

PROBLEM: Low Bond Precision on C-C Bonds .... 0.02333 Ang.

RESPONSE: The crystals all showed strong diffuse scattering in the form of diffuse Bragg peaks and scattering between them. As the model does not account for this type of disorder this results in low bond precision.

Supplementary Methods

Synthesis of H_4bptc

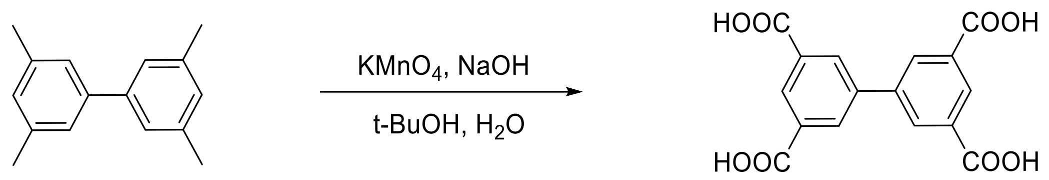

The synthesis of H_4bptc was carried out according to reported procedure with slight modifications[34]. 3,3'5,5'-tetramethylbiphenyl (5.0 g, 0.023 mol), NaOH (2.0 g, 0.05 mol) were mixed in t-BuOH/H_2O (100/100 mL) with stirring at 50 °C. $KMnO_4$ (43.0 g, 0.27 mol) was added in portions over one week. The temperature was subsequently increased to 70 °C and kept for 2 days. The mixture was filter when hot and the clear filtrate was added into 100 mL 6M HCl. White solid was obtained upon filtration. The crude product was recrystallized from DMF (~100 mL) to give pure H_4bptc with a yield 82%. ^{1}H NMR (400 MHz, DMSO-d^6): δ= 13.50 (4H, COOH), 8.51 (2H, Ar-H), 8.42 (4H, Ar-H).

Synthesis of H_4abtc

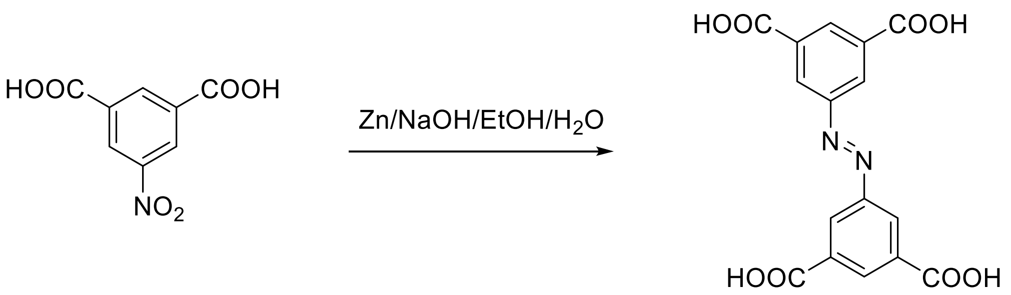

H_4abtc was synthesized according to reported procedure with slight modifications[35,36]. 5-nitroisophthalic acid (2.1 g, 0.01 mol), NaOH (3.2 g, 0.08 mol), Zinc powder (2.1 g, 0.04 mol) were mixed in ethanol/H_2O (50/20 mL). The mixture was kept under refluxing for 12 hours before

cooled to room temperature. Yellow solid was obtained through vacuum filtration which was then dissolved in 80 mL 1M NaOH solution. Upon filtration, the filtrate was acidified with 6 M HCl to get orange solid. The crude product was recrystallized from DMF to give pure H_4abtc as orange solid (1.3 g, yield: 73%). ^{1}H NMR (400 MHz, DMSO-d^6): δ= 13.38 (4H, COOH), 8.58-8.61 (6H, Ar-H).

<u>Synthesis of H_4tptc-(Me)$_2$</u>[37,38]

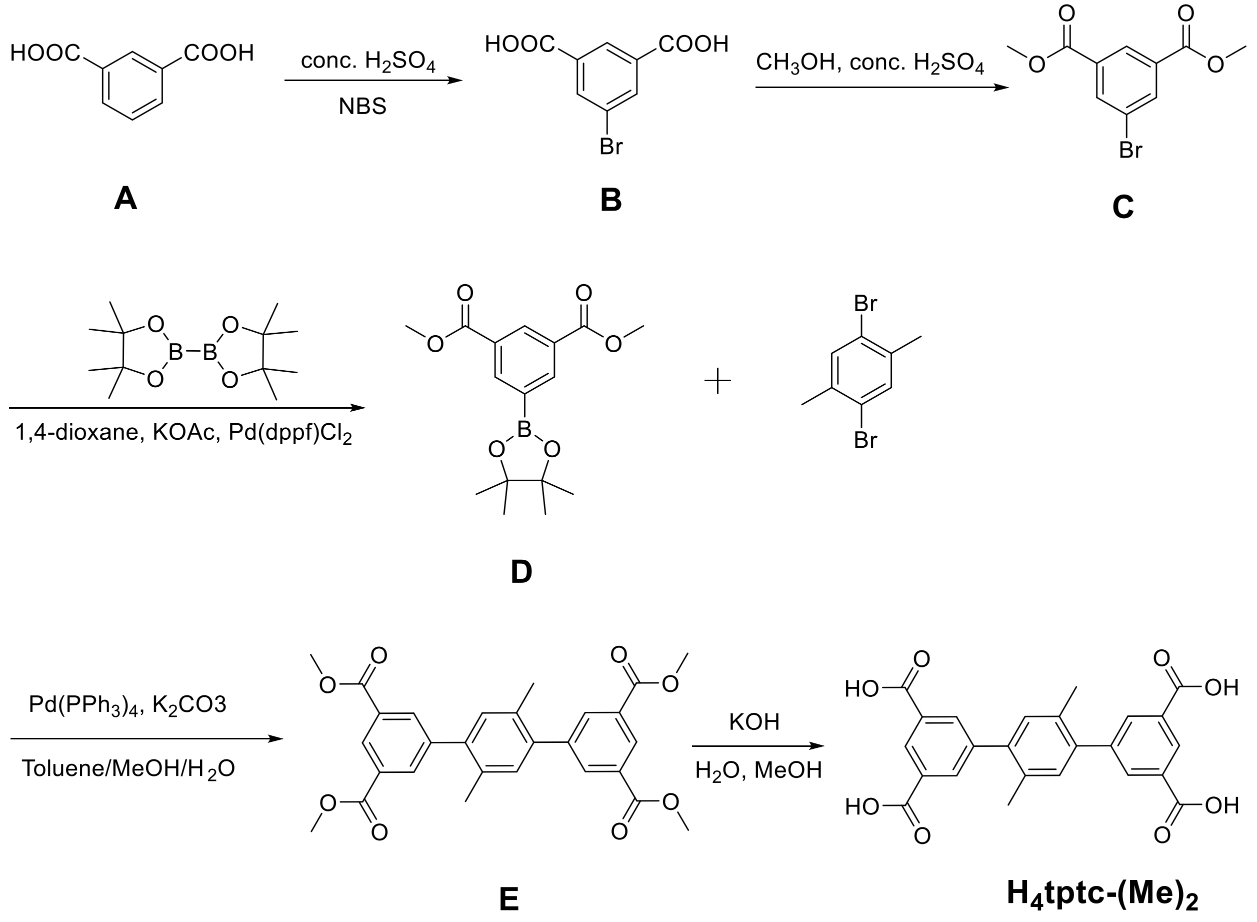

A to **B**: A (14.0 g, 0.084 mol) was dissolved in 40 mL conc. H_2SO_4 at 60 °C under stirring. N-Bromosuccinimide (NBS, 16 g, 0.090 mol) was added to the reaction in portions over 1 hour and the mixture was stirred at 60 °C for an additional hour. After the reaction was completed, the

mixture was poured into 200 g crushed ice and stirred at room temperature for 1 hour. Light yellow solid was obtained by filtration and washed with DI water (100 mL). The crude product was recrystallized from ethyl acetate to give pure **B** (12 g, 59 % yield).

B to **C**: **B** (8 g, 0.033 mol) was dissolved in 120 mL methanol under stirring and 6 mL conc. H_2SO_4 was subsequently added to the mixture. The reaction was heated to reflux for 24 hours. After cooling down to room temperature the solvent was removed through rotovap. The obtained solid was dispersed in 40 mL DI water and the aqueous phase was extracted by dichloromethane (40 mL × 3). The combined organic layer was washed with saturated $NaHCO_3$ and brine and dried over $MgSO_4$. Crude product was obtained upon removal of the solvent which was recrystallized from methanol to give pure **C** (8.3 g, 91 % yield).

C to **D**: **C** (3.3 g, 0.012 mol), bis(pinacolato)diboron (3.4 g, 0.013 mol), dried potassium acetate (3.6 g), [1,1'-Bis(diphenylphosphino)ferrocene]palladium(II) dichloride (Pd(dppf)Cl_2, 0.2 g, 0.27 mol) were mixed in a 250 mL flask and evacuated for 1 hour before 100 mL dried, degassed 1,4-dioxane was added. The reaction was stirred at 80 °C under nitrogen atmosphere for 24 hours. After the reaction was completed, organic solvent was removed by rotovap and the remained solid was extracted with dichloromethane. The crude product was purified through a silica plug with a eluent of petroleum ether: ethyl acetate= 8: 1 to give pure **D** as a white solid (3.5 g, 90 % yield).

D to **E**: **D** (2.0 g, 6.3 mmol), 2,5-dibromo-p-xylene (0.6 g, 2.3 mmol), tetrakis (triphenylphosphine) palladium(0) (150 mg, 0.15 mmol) and potassium carbonate (760 mg, 5.5 mmol) were mixed in a 100 mL flask and evacuated for 1 hour before 40 mL degassed toluene-methanol-water (v:v:v = 20:10:10) mixed solvent was added. The reaction was heated to reflux for 3 days. The reaction was cooled to room temperature when it was completed and organic solvent was removed by rotary evaporation. 50 mL DI water was added to the residue which was extracted by dichloromethane

for 3 times with 50 mL each time. The organic phase was combined and washed with water and brine and dried over anhydrous $MgSO_4$. After removal of organic solvent, the crude product was washed with a mixed solvent of acetone/dichloromethane (v/v = 9/1) several times (10 mL × 5) to give pure compound **E** (0.68 g, 60% yield).

E to H_4tptc-(Me)$_2$: **E** (0.5 g, 1 mmol) was dissolved in a mixture of 30 mL THF and 30 mL 3M KOH aqueous solution. The mixture was heated to reflux for 12 hours. After removal of THF, the remaining clear solution was added dropwise to excess 6 M HCl solution. The white precipitate was collected by centrifuge and washed with DI water until it is neutral. The resultant solid was dried at 80 °C under vacuum to give pure H_4-tptc-(Me)$_2$ (0.4 g, 92% yield). ^{1}H NMR (400 MHz, DMSO-d^6): δ= 13.43 (4H, COOH), 8.48 (2H, Ar-H), 8.17 (4H, Ar-H), 7.26 (2H, Ar-H), 2.23 (6H, CH_3).

Synthesis of UiO-66 and UiO-67

UiO-66 and UiO-67 were synthesized according to reported literature[39]. Phase purity was confirmed by PXRD analysis.

Synthesis of Y-fum

Y-fum was synthesized according to reported literature[40]. Phase purity was confirmed by PXRD analysis.

Supplementary References

1 Altomare, A. *et al. EXPO2013: A kit of tools for phasing crystal structures from powder data*. Vol. 46 (2013).

2 A., Y. R. *The Rietveld Method. IUCr Book Serials*. (Oxford University Press, 1993).

3 Herm, Z. R. *et al.* Separation of Hexane Isomers in a Metal-Organic Framework with Triangular Channels. *Science* **340**, 960-964 (2013).

4 Peralta, D., Chaplais, G., Simon-Masseron, A., Barthelet, K. & Pirngruber, G. D. Separation of C6 Paraffins Using Zeolitic Imidazolate Frameworks: Comparison with Zeolite 5A. *Industrial & Engineering Chemistry Research* **51**, 4692-4702 (2012).

5 Burgess, S. A. *et al.* Improved Catalytic Activity and Stability of a Palladium Pincer Complex by Incorporation into a Metal–Organic Framework. *J. Am. Chem. Soc.* **138**, 1780-1783 (2016).

6 Gutov, O. V. *et al.* Water-Stable Zirconium-Based Metal–Organic Framework Material with High-Surface Area and Gas-Storage Capacities. *Chem. Eur. J.* **20**, 12389-12393 (2014).

7 Kalidindi, S. B. *et al.* Chemical and Structural Stability of Zirconium-based Metal–Organic Frameworks with Large Three-Dimensional Pores by Linker Engineering. *Angewandte Chemie International Edition* **54**, 221-226 (2015).

8 Liu, T.-F. *et al.* Topology-Guided Design and Syntheses of Highly Stable Mesoporous Porphyrinic Zirconium Metal–Organic Frameworks with High Surface Area. *J. Am. Chem. Soc.* **137**, 413-419 (2015).

9 Deria, P. *et al.* Ultraporous, Water Stable, and Breathing Zirconium-Based Metal–Organic Frameworks with ftw Topology. *J. Am. Chem. Soc.* **137**, 13183-13190 (2015).

10 Wang, T. C. *et al.* Ultrahigh Surface Area Zirconium MOFs and Insights into the Applicability of the BET Theory. *J. Am. Chem. Soc.* **137**, 3585-3591 (2015).

11 Lin, Q. *et al.* New Heterometallic Zirconium Metalloporphyrin Frameworks and Their Heteroatom-Activated High-Surface-Area Carbon Derivatives. *J. Am. Chem. Soc.* **137**, 2235-2238 (2015).

12 Zheng, J., Wu, M., Jiang, F., Su, W. & Hong, M. Stable porphyrin Zr and Hf metal-organic frameworks featuring 2.5 nm cages: high surface areas, SCSC transformations and catalyses. *Chem. Sci.* **6**, 3466-3470 (2015).

13 Wei, Z. *et al.* Rigidifying Fluorescent Linkers by Metal–Organic Framework Formation for Fluorescence Blue Shift and Quantum Yield Enhancement. *Journal of the American Chemical Society* **136**, 8269-8276 (2014).

14 Morris, W. *et al.* Synthesis, Structure, and Metalation of Two New Highly Porous Zirconium Metal–Organic Frameworks. *Inorg. Chem.* **51**, 6443-6445 (2012).

15 Zhang, Q. *et al.* Piezofluorochromic Metal–Organic Framework: A Microscissor Lift. *J. Am. Chem. Soc.* **137**, 10064-10067 (2015).

16 Wang, R., Bragaglia, V., Boschker, J. E. & Calarco, R. Intermixing during Epitaxial Growth of van der Waals Bonded Nominal GeTe/Sb2Te3 Superlattices. *Cryst. Growth Des.* **16**, 3596-3601 (2016).

17 Feng, D. *et al.* Zirconium-Metalloporphyrin PCN-222: Mesoporous Metal–Organic Frameworks with Ultrahigh Stability as Biomimetic Catalysts. *Angew. Chem. Int. Ed.* **51**, 10307-10310 (2012).

18 Mondloch, J. E. *et al.* Vapor-Phase Metalation by Atomic Layer Deposition in a Metal–Organic Framework. *J. Am. Chem. Soc.* **135**, 10294-10297 (2013).

19 Wang, B. *et al.* Highly Stable Zr(IV)-Based Metal–Organic Frameworks for the Detection and Removal of Antibiotics and Organic Explosives in Water. *J. Am. Chem. Soc.* **138**, 6204-6216 (2016).

20 Jiang, H.-L. *et al.* An Exceptionally Stable, Porphyrinic Zr Metal–Organic Framework Exhibiting pH-Dependent Fluorescence. *J. Am. Chem. Soc.* **135**, 13934-13938 (2013).

21 Xu, L. *et al.* Highly Stable Mesoporous Zirconium Porphyrinic Frameworks with Distinct Flexibility. *Chem. Eur. J.* **22**, 6268-6276 (2016).

22 Feng, D. *et al.* A Highly Stable Porphyrinic Zirconium Metal–Organic Framework with shp-a Topology. *J. Am. Chem. Soc.* **136**, 17714-17717 (2014).

23 Gallagher, A. T. *et al.* Dioxygen binding at a four-coordinate cobaltous porphyrin site in a metal-organic framework: structural, EPR, and O2 adsorption analysis. *Inorg. Chem. Front.* **3**, 536-540 (2016).

24 Anderson, J. S., Gallagher, A. T., Mason, J. A. & Harris, T. D. A Five-Coordinate Heme Dioxygen Adduct Isolated within a Metal–Organic Framework. *J. Am. Chem. Soc.* **136**, 16489-16492 (2014).

25 Cui, H. *et al.* A stable and porous iridium(iii)-porphyrin metal-organic framework: synthesis, structure and catalysis. *CrystEngComm* **18**, 2203-2209 (2016).

26 Feng, D. *et al.* Construction of Ultrastable Porphyrin Zr Metal–Organic Frameworks through Linker Elimination. *J. Am. Chem. Soc.* **135**, 17105-17110 (2013).

27 Furukawa, H. *et al.* Water Adsorption in Porous Metal–Organic Frameworks and Related Materials. *Journal of the American Chemical Society* **136**, 4369-4381 (2014).

28 Zhang, M. *et al.* Symmetry-Guided Synthesis of Highly Porous Metal–Organic Frameworks with Fluorite Topology. *Angew. Chem. Int. Ed.* **53**, 815-818 (2014).

29 Wang, S. *et al.* A Zr metal-organic framework based on tetrakis(4-carboxyphenyl) silane and factors affecting the hydrothermal stability of Zr-MOFs. *Dalton Trans.* **44**, 8049-8061 (2015).

30 Kresse, G. & Furthmüller, J. Efficient iterative schemes for ab initio total-energy calculations using a plane-wave basis set. *Phys. Rev. B* **54**, 11169-11186 (1996).

31 Kresse, G. & Joubert, D. From ultrasoft pseudopotentials to the projector augmented-wave method. *Phys. Rev. B* **59**, 1758-1775 (1999).

32 Henkelman, G., Uberuaga, B. P. & Jónsson, H. A climbing image nudged elastic band method for finding saddle points and minimum energy paths. *J. Chem. Phys.* **113**, 9901-9904 (2000).

33 Henkelman, G. & Jónsson, H. Improved tangent estimate in the nudged elastic band method for finding minimum energy paths and saddle points. *J. Chem. Phys.* **113**, 9978-9985 (2000).

34 Huang, Y.-G. *et al.* Thermally Induced Intra-Carboxyl Proton Shuttle in a Molecular Rack-and-Pinion Cascade Achieving Macroscopic Crystal Deformation. *Angew. Chem. Int. Ed.* **55**, 14628-14632 (2016).

35 Wang, S., Wang, X., Li, L. & Advincula, R. C. Design, Synthesis, and Photochemical Behavior of Poly(benzyl ester) Dendrimers with Azobenzene Groups throughout Their Architecture. *The Journal of Organic Chemistry* **69**, 9073-9084 (2004).

36 Wang, X.-S. *et al.* Metal−Organic Frameworks Based on Double-Bond-Coupled Di-Isophthalate Linkers with High Hydrogen and Methane Uptakes. *Chem. Mater.* **20**, 3145-3152 (2008).

37 Xia, T. *et al.* Microporous metal-organic frameworks with suitable pore spaces for acetylene storage and purification. *Micro. Meso. Mater.* **215**, 109-115 (2015).

38 Lin, X. *et al.* High Capacity Hydrogen Adsorption in Cu(II) Tetracarboxylate Framework Materials: The Role of Pore Size, Ligand Functionalization, and Exposed Metal Sites. *J. Am. Chem. Soc.* **131**, 2159-2171 (2009).

39 Cavka, J. H. *et al.* A New Zirconium Inorganic Building Brick Forming Metal Organic Frameworks with Exceptional Stability. *Journal of the American Chemical Society* **130**, 13850-13851 (2008).

40 Assen, A. H. *et al.* Ultra-Tuning of the Rare-Earth fcu-MOF Aperture Size for Selective Molecular Exclusion of Branched Paraffins. *Angew. Chem. Int. Ed.* **54**, 14353-14358 (2015).
