# Supplementary material for: Topologically guided tuning of Zr-MOF pore structures for highly selective separation of C6 alkane isomers
Source: Nat Commun. 2018 May 1;9:1745. doi: 10.1038/s41467-018-04152-5 (PMC5931593; doi:10.1038/s41467-018-04152-5)
Supplement: Supplementary file 3 — Description of Additional Supplementary Files [file 41467_2018_4152_MOESM3_ESM.pdf]

Description of Additional Supplementary Files

File Name: Supplementary Data 1
Description: Cif file of Compound 1.

File Name: Supplementary Data 2
Description: Checkcif file of Compound 1.

File Name: Supplementary Data 3
Description: Cif file of Compound 2.

File Name: Supplementary Data 4
Description: Checkcif file of Compound 2.

File Name: Supplementary Data 5
Description: Cif file of Compound 3.

File Name: Supplementary Data 6
Description: Checkcif file of Compound 3.

File Name: Supplementary Data 7
Description: All references, topologies and geometrical parameters for the 145+56 structures listed in Supplementary Tables 4-6 and Fig. 2a.
