# Supplementary material for: Topologically guided tuning of Zr-MOF pore structures for highly selective separation of C6 alkane isomers
Source: Nat Commun. 2018 May 1;9:1745. doi: 10.1038/s41467-018-04152-5 (PMC5931593; doi:10.1038/s41467-018-04152-5)
Supplement: Supplementary file 5 — Supplementary Data 2 [file 41467_2018_4152_MOESM5_ESM.pdf]

No syntax errors found.
Please wait while processing ....

CIF dictionary
Interpreting this report

Datablock: topas_cif_out

Bond precision:	C-C = 0.0028 A		Wavelength=1.54060
Cell:	a=24.3596(3)	b=24.3596(3)	c=24.3596(3)
	alpha=90	beta=90	gamma=90
Temperature:	295 K		

	Calculated	Reported
Volume	14454.8(5)	14454.8(5)
Space group	I m -3	Im-3
Hall group	-I 2 2 3	?
Moiety formula	C24 H9 O16 Zr3	?
Sum formula	C24 H9 O16 Zr3	C48 H18 O32 Zr6
Mr	826.97	1653.34
Dx,g cm-3	1.520	0.000
Z	16	8
Mu (mm-1)	7.364	0.000
F000	6416.0	0.0
F000'	6426.41	
h,k,lmax	18,18,18	
Nref	592	
Tmin,Tmax		
Tmin'		

Correction method= Not given

Data completeness= 0.000 Theta(max)=

R(reflections)= wR2(reflections)=

S = Npar=

The following ALERTS were generated. Each ALERT has the format
test-name_ALERT_alert-type_alert-level.
Click on the hyperlinks for more details of the test.

Alert level A

GEOM001_ALERT_1_A _geom_bond_atom_site_label_1 is missing
Label identifying the atom site 1.
GEOM003_ALERT_1_A _geom_bond_distance is missing
Distance between atom sites 1 and 2.
GEOM006_ALERT_1_A _geom_angle_atom_site_label_2 is missing
Label identifying the atom site 2.
GEOM007_ALERT_1_A _geom_angle_atom_site_label_3 is missing
Label identifying the atom site 3.
PLAT602_ALERT_2_A VERY LARGE Solvent Accessible VOID(S) in Structure ! Info

Alert level B

POWD002_ALERT_1_B _refine_ls_goodness_of_fit_all is missing (this is chi, i.e.
the square root of 'chi squared'). This should be
present for a powder diffraction study.
POWD004_ALERT_1_B No 'Bragg' R factor has been given. Please supply a value
for _refine_ls_R_factor_all [R(F)], refine_ls_R_Fsqd_factor
[R(F^2)] or _refine_ls_R_I_factor [R(I)].

Alert level C

REFI015_ALERT_1_C _refine_ls_shift/su_max is missing
Maximum shift/s.u. ratio after final refinement cycle.
The following tests will not be performed
SHFSU_01
CRYSC01_ALERT_1_C No recognised colour has been given for crystal colour.
PLAT193_ALERT_1_C Cell and Diffraction Temperatures Differ by .... 2 Degree
PLAT241_ALERT_2_C High 'MainMol' Ueq as Compared to Neighbors of C5 Check
PLAT241_ALERT_2_C High 'MainMol' Ueq as Compared to Neighbors of C10 Check
PLAT242_ALERT_2_C Low 'MainMol' Ueq as Compared to Neighbors of Zr1 Check
PLAT369_ALERT_2_C Long C(sp2)-C(sp2) Bond C2 - C5 .. 1.54 Ang.
And 3 other PLAT369 Alerts

PLAT369_ALERT_2_C Long C(sp2)-C(sp2) Bond C4 - C4_a .. 1.54 Ang.
PLAT369_ALERT_2_C Long C(sp2)-C(sp2) Bond C6 - C6_b .. 1.55 Ang.

```
PLAT369_ALERT_2_C Long    C(sp2)-C(sp2) Bond  C8      -   C10     ..        1.54 Ang.
```

Alert level G

```
PLAT004_ALERT_5_G Polymeric Structure Found with Maximum Dimension           3 Info
PLAT045_ALERT_1_G Calculated and Reported Z Differ by a Factor ...        2.00 Check
PLAT092_ALERT_4_G Check: Wavelength given is not Cu,Ga,Mo,Ag,In Ka    1.54060 Ang.
PLAT164_ALERT_4_G Nr. of Refined C-H H-Atoms in Heavy-Atom Struct.           4 Note
PLAT860_ALERT_3_G Number of Least-Squares Restraints .............          22 Note
```

```
   5 ALERT level A = Most likely a serious problem - resolve or explain
   2 ALERT level B = A potentially serious problem, consider carefully
  10 ALERT level C = Check. Ensure it is not caused by an omission or oversight
   5 ALERT level G = General information/check it is not something unexpected

  10 ALERT type 1 CIF construction/syntax error, inconsistent or missing data
   8 ALERT type 2 Indicator that the structure model may be wrong or deficient
   1 ALERT type 3 Indicator that the structure quality may be low
   2 ALERT type 4 Improvement, methodology, query or suggestion
   1 ALERT type 5 Informative message, check
```

It is advisable to attempt to resolve as many as possible of the alerts in all categories. Often the minor alerts point to easily fixed oversights, errors and omissions in your CIF or refinement strategy, so attention to these fine details can be worthwhile. In order to resolve some of the more serious problems it may be necessary to carry out additional measurements or structure refinements. However, the purpose of your study may justify the reported deviations and the more serious of these should normally be commented upon in the discussion or experimental section of a paper or in the "special_details" fields of the CIF. checkCIF was carefully designed to identify outliers and unusual parameters, but every test has its limitations and alerts that are not important in a particular case may appear. Conversely, the absence of alerts does not guarantee there are no aspects of the results needing attention. It is up to the individual to critically assess their own results and, if necessary, seek expert advice.

Publication of your CIF in IUCr journals

A basic structural check has been run on your CIF. These basic checks will be run on all CIFs submitted for publication in IUCr journals (*Acta Crystallographica*, *Journal of Applied Crystallography*, *Journal of Synchrotron Radiation*); however, if you intend to submit to *Acta Crystallographica Section C* or *E* or *IUCrData*, you should make sure that full publication checks are run on the final version of your CIF prior to submission.

Publication of your CIF in other journals

Please refer to the *Notes for Authors* of the relevant journal for any special instructions relating to CIF submission.

PLATON version of 27/03/2017; check.def file version of 24/03/2017

Datablock topas_cif_out - ellipsoid plot

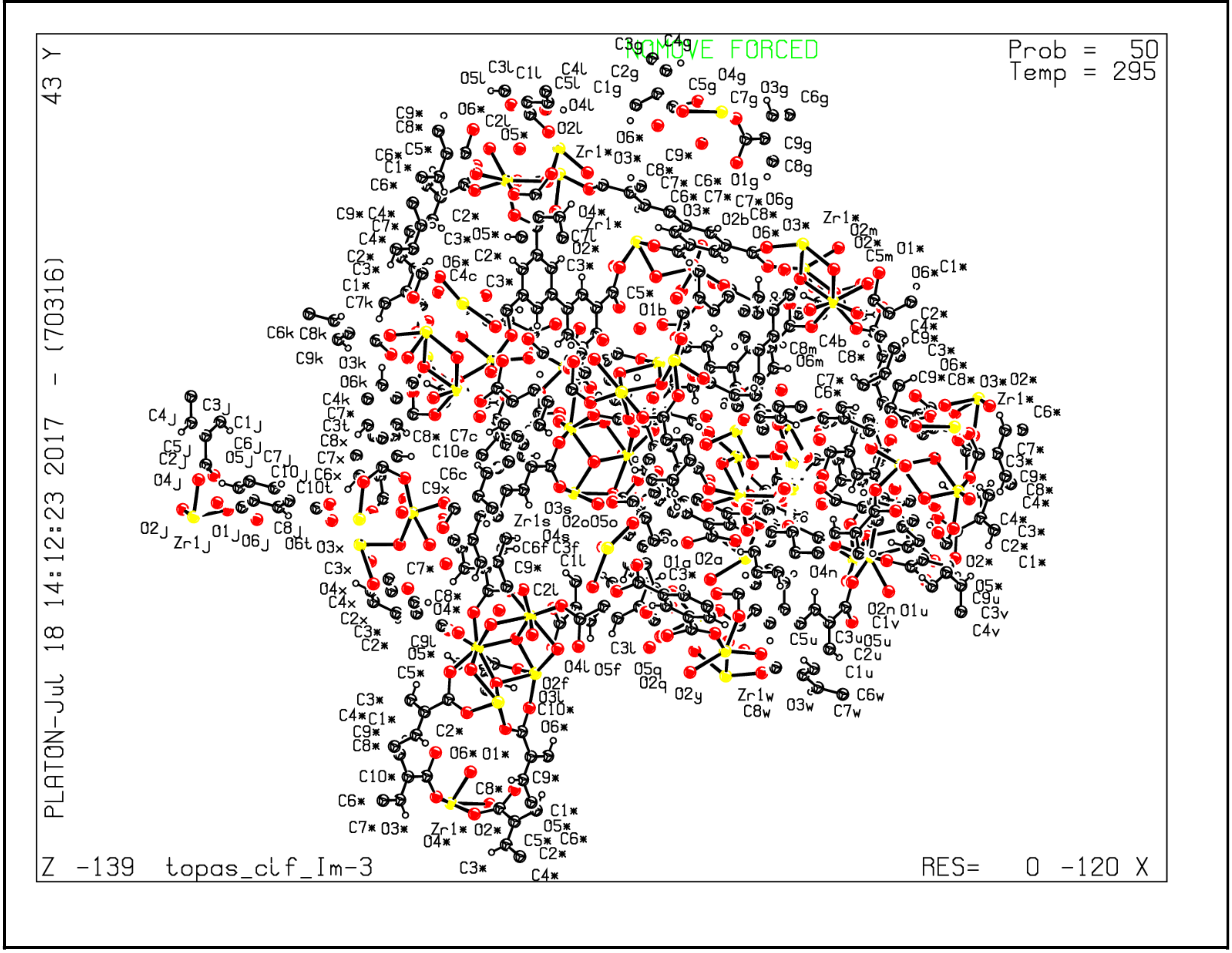

[Download CIF editor (publCIF) from the IUCr](#)
[Download CIF editor (enCIFer) from the CCDC](#)
[Test a new CIF entry](#)
