# Supplementary material for: Topologically guided tuning of Zr-MOF pore structures for highly selective separation of C6 alkane isomers
Source: Nat Commun. 2018 May 1;9:1745. doi: 10.1038/s41467-018-04152-5 (PMC5931593; doi:10.1038/s41467-018-04152-5)

checkCIF/PLATON report

Structure factors have been supplied for datablock(s) twin5

THIS REPORT IS FOR GUIDANCE ONLY. IF USED AS PART OF A REVIEW PROCEDURE FOR PUBLICATION, IT SHOULD NOT REPLACE THE EXPERTISE OF AN EXPERIENCED CRYSTALLOGRAPHIC REFEREE.

No syntax errors found. CIF dictionary Interpreting this report

Datablock: twin5

```
Bond precision:  C-C = 0.0372 A          Wavelength=0.77490

Cell:           a=25.4692(11)    b=36.3589(15)      c=21.5275(9)
                alpha=90         beta=122.260(2)    gamma=90
Temperature:    100 K

                Calculated                   Reported
Volume          16857.9(13)                  16857.8(13)
Space group     C 2/m                        C 2/m
Hall group      -C 2y                        -C 2y
Moiety formula  C32 H12 N4 O32 Zr6           ?
Sum formula     C32 H12 N4 O32 Zr6           C32 H20 N4 O32 Zr6
Mr              1511.78                      1519.84
Dx,g cm-3       1.191                        1.198
Z               8                            8
Mu (mm-1)       0.964                        0.965
F000            5824.0                       5888.0
F000'           5740.97
h,k,lmax        30,43,25                     30,43,25
Nref            15772                        22502
Tmin,Tmax       0.977,0.981                  0.612,0.981
Tmin'           0.882

Correction method= # Reported T Limits: Tmin=0.612 Tmax=0.981
AbsCorr = MULTI-SCAN

Data completeness= 1.427          Theta(max)= 27.868

R(reflections)= 0.0877( 15930)    wR2(reflections)= 0.2802( 22502)

S = 1.052                  Npar= 678
```

The following ALERTS were generated. Each ALERT has the format
test-name_ALERT_alert-type_alert-level.
Click on the hyperlinks for more details of the test.

Alert level A

PLAT602_ALERT_2_A VERY LARGE Solvent Accessible VOID(S) in Structure ! Info

Author Response: There is a large solvent channel in the structure but as a result of the twinning no clear solvent molecules could be found.

Alert level B

PLAT213_ALERT_2_B Atom C8 has ADP max/min Ratio ..... 4.6 prolat

Author Response: The twinning has had an adverse effect of the data which require significant displacement parameter restraints. Even tighter restraints would be required, so it was decided to leave it as it is.

PLAT342_ALERT_3_B Low Bond Precision on C-C Bonds .... 0.03719 Ang.

Author Response: The twinning has had an adverse effect of the data.

PLAT430_ALERT_2_B Short Inter D...A Contact O9 .. O26 .. 2.70 Ang.

Author Response: Ligand carboxylate groups are in close approximate to mu3 hydroxides in the cluster or neighbouring carboxylate groups.

PLAT430_ALERT_2_B Short Inter D...A Contact O9 .. O25 .. 2.79 Ang.

Author Response: Ligand carboxylate groups are in close approximate to mu3 hydroxides in the cluster or neighbouring carboxylate groups.

PLAT430_ALERT_2_B Short Inter D...A Contact O14 .. O25 .. 2.70 Ang.

Author Response: Ligand carboxylate groups are in close approximate to mu3 hydroxides in the cluster or neighbouring carboxylate groups.

PLAT430_ALERT_2_B Short Inter D...A Contact O18 .. O24 .. 2.68 Ang.

Author Response: Ligand carboxylate groups are in close approximate to mu3 hydroxides in the cluster or neighbouring carboxylate groups.

PLAT430_ALERT_2_B Short Inter D...A Contact O24 .. O25 .. 2.63 Ang.

Author Response: Ligand carboxylate groups are in close approximate to mu3 hydroxides in the cluster or neighbouring carboxylate groups.

```
PLAT910_ALERT_3_B Missing # of FCF Reflection(s) Below Theta(Min)          13 Note
```

Author Response: The unit cell is reasonable large and these low angle reflections are probably missing due to the beamstop.

Alert level C

```
DIFMX02_ALERT_1_C  The maximum difference density is > 0.1*ZMAX*0.75
            The relevant atom site should be identified.
PLAT041_ALERT_1_C Calc. and Reported SumFormula    Strings  Differ     Please Check
PLAT043_ALERT_1_C Calculated and Reported Mol. Weight Differ by ..       8.06 Check
PLAT068_ALERT_1_C Reported F000 Differs from Calcd (or Missing)...     Please Check
PLAT084_ALERT_3_C High wR2 Value (i.e. > 0.25) ...................       0.28 Report
PLAT097_ALERT_2_C Large Reported Max.  (Positive) Residual Density       3.04 eA-3
PLAT213_ALERT_2_C Atom O5              has ADP max/min Ratio .....        3.5 prolat
```

Author Response: The twinning has had an adverse effect of the data which require significant displacement parameter restraints. Even tighter restraints would be required, so it was decided to leave it as it is.

```
PLAT213_ALERT_2_C Atom O8              has ADP max/min Ratio .....        3.4 oblate
```

Author Response: The twinning has had an adverse effect of the data which require significant displacement parameter restraints. Even tighter restraints would be required, so it was decided to leave it as it is.

```
PLAT213_ALERT_2_C Atom O13             has ADP max/min Ratio .....        3.2 prolat
```

Author Response: The twinning has had an adverse effect of the data which require significant displacement parameter restraints. Even tighter restraints would be required, so it was decided to leave it as it is.

```
PLAT220_ALERT_2_C Non-Solvent Resd 1   C   Ueq(max)/Ueq(min) Range        3.4 Ratio
PLAT220_ALERT_2_C Non-Solvent Resd 1   O   Ueq(max)/Ueq(min) Range        5.8 Ratio
PLAT241_ALERT_2_C High   'MainMol' Ueq as Compared to Neighbors of         O1 Check
PLAT241_ALERT_2_C High   'MainMol' Ueq as Compared to Neighbors of         O2 Check
PLAT241_ALERT_2_C High   'MainMol' Ueq as Compared to Neighbors of         O4 Check
PLAT241_ALERT_2_C High   'MainMol' Ueq as Compared to Neighbors of         O5 Check
PLAT241_ALERT_2_C High   'MainMol' Ueq as Compared to Neighbors of         O7 Check
PLAT241_ALERT_2_C High   'MainMol' Ueq as Compared to Neighbors of         O8 Check
PLAT241_ALERT_2_C High   'MainMol' Ueq as Compared to Neighbors of        O10 Check
PLAT241_ALERT_2_C High   'MainMol' Ueq as Compared to Neighbors of        O13 Check
PLAT241_ALERT_2_C High   'MainMol' Ueq as Compared to Neighbors of        O20 Check
PLAT241_ALERT_2_C High   'MainMol' Ueq as Compared to Neighbors of         N1 Check
PLAT242_ALERT_2_C Low    'MainMol' Ueq as Compared to Neighbors of        Zr1 Check
PLAT242_ALERT_2_C Low    'MainMol' Ueq as Compared to Neighbors of        Zr2 Check
PLAT242_ALERT_2_C Low    'MainMol' Ueq as Compared to Neighbors of        Zr3 Check
PLAT242_ALERT_2_C Low    'MainMol' Ueq as Compared to Neighbors of        Zr4 Check
PLAT242_ALERT_2_C Low    'MainMol' Ueq as Compared to Neighbors of        Zr5 Check
PLAT242_ALERT_2_C Low    'MainMol' Ueq as Compared to Neighbors of        Zr6 Check
PLAT242_ALERT_2_C Low    'MainMol' Ueq as Compared to Neighbors of        Zr7 Check
PLAT790_ALERT_4_C Centre of Gravity not Within Unit Cell: Resd.  #          1 Note
              C32 H12 N4 O32 Zr6
```

```
PLAT905_ALERT_3_C Negative K value in the Analysis of Variance ...     -0.253 Report
PLAT911_ALERT_3_C Missing # FCF Refl Between THmin & STh/L=  0.600       808 Report
PLAT918_ALERT_3_C Reflection(s) with I(obs) much Smaller I(calc) .        11 Check
```

Alert level G

```
FORMU01_ALERT_2_G  There is a discrepancy between the atom counts in the
            _chemical_formula_sum and the formula from the _atom_site* data.
            Atom count from _chemical_formula_sum:C32 H20 N4 O32 Zr6
            Atom count from the _atom_site data:  C32 H12 N4 O32 Zr6
ABSMU01_ALERT_1_G  Calculation of _exptl_absorpt_correction_mu
                not performed for this radiation type.
CELLZ01_ALERT_1_G Difference between formula and atom_site contents detected.
CELLZ01_ALERT_1_G WARNING: H atoms missing from atom site list. Is this intentional?
           From the CIF: _cell_formula_units_Z    8
           From the CIF: _chemical_formula_sum  C32 H20 N4 O32 Zr6
           TEST: Compare cell contents of formula and atom_site data

           atom    Z*formula  cif sites diff
           C        256.00    256.00    0.00
           H        160.00     96.00   64.00
           N         32.00     32.00    0.00
           O        256.00    256.00    0.00
           Zr        48.00     48.00    0.00
PLAT003_ALERT_2_G Number of Uiso or Uij Restrained non-H Atoms ...        77 Report
PLAT004_ALERT_5_G Polymeric Structure Found with Maximum Dimension         3 Info
PLAT072_ALERT_2_G SHELXL First  Parameter in WGHT  Unusually Large      0.15 Report
PLAT083_ALERT_2_G SHELXL Second Parameter in WGHT  Unusually Large    545.37 Why ?
PLAT128_ALERT_4_G Alternate Setting for Input Space Group     C2/m      I2/m Note
PLAT174_ALERT_4_G The CIF-Embedded .res File Contains FLAT Records         1 Report
PLAT186_ALERT_4_G The CIF-Embedded .res File Contains ISOR Records         2 Report
PLAT764_ALERT_4_G Overcomplete CIF Bond List Detected (Rep/Expd) .      1.40 Ratio
PLAT860_ALERT_3_G Number of Least-Squares Restraints .............       468 Note
PLAT870_ALERT_4_G ALERTS Related to Twinning Effects Suppressed ..         ! Info
PLAT912_ALERT_4_G Missing # of FCF Reflections Above STh/L=  0.600        65 Note
PLAT913_ALERT_3_G Missing # of Very Strong Reflections in FCF ....         2 Note
PLAT931_ALERT_5_G Found Twin Law ( 0 0  1)[       ] Estimated BASF      0.21 Check
PLAT933_ALERT_2_G Number of OMIT Records in Embedded .res File ...        11 Note
```

---

 1 **ALERT level A** = Most likely a serious problem - resolve or explain
 8 **ALERT level B** = A potentially serious problem, consider carefully
32 **ALERT level C** = Check. Ensure it is not caused by an omission or oversight
18 **ALERT level G** = General information/check it is not something unexpected

```
 7 ALERT type 1 CIF construction/syntax error, inconsistent or missing data
35 ALERT type 2 Indicator that the structure model may be wrong or deficient
 8 ALERT type 3 Indicator that the structure quality may be low
 7 ALERT type 4 Improvement, methodology, query or suggestion
 2 ALERT type 5 Informative message, check
```

It is advisable to attempt to resolve as many as possible of the alerts in all categories. Often the minor alerts point to easily fixed oversights, errors and omissions in your CIF or refinement strategy, so attention to these fine details can be worthwhile. In order to resolve some of the more serious problems it may be necessary to carry out additional measurements or structure refinements. However, the purpose of your study may justify the reported deviations and the more serious of these should normally be commented upon in the discussion or experimental section of a paper or in the "special_details" fields of the CIF. checkCIF was carefully designed to identify outliers and unusual parameters, but every test has its limitations and alerts that are not important in a particular case may appear. Conversely, the absence of alerts does not guarantee there are no aspects of the results needing attention. It is up to the individual to critically assess their own results and, if necessary, seek expert advice.

Publication of your CIF in IUCr journals

A basic structural check has been run on your CIF. These basic checks will be run on all CIFs submitted for publication in IUCr journals (*Acta Crystallographica*, *Journal of Applied Crystallography*, *Journal of Synchrotron Radiation*); however, if you intend to submit to *Acta Crystallographica Section C* or *E* or *IUCrData*, you should make sure that full publication checks are run on the final version of your CIF prior to submission.

Publication of your CIF in other journals

Please refer to the *Notes for Authors* of the relevant journal for any special instructions relating to CIF submission.

PLATON version of 24/11/2016; check.def file version of 23/11/2016

Datablock twin5 - ellipsoid plot

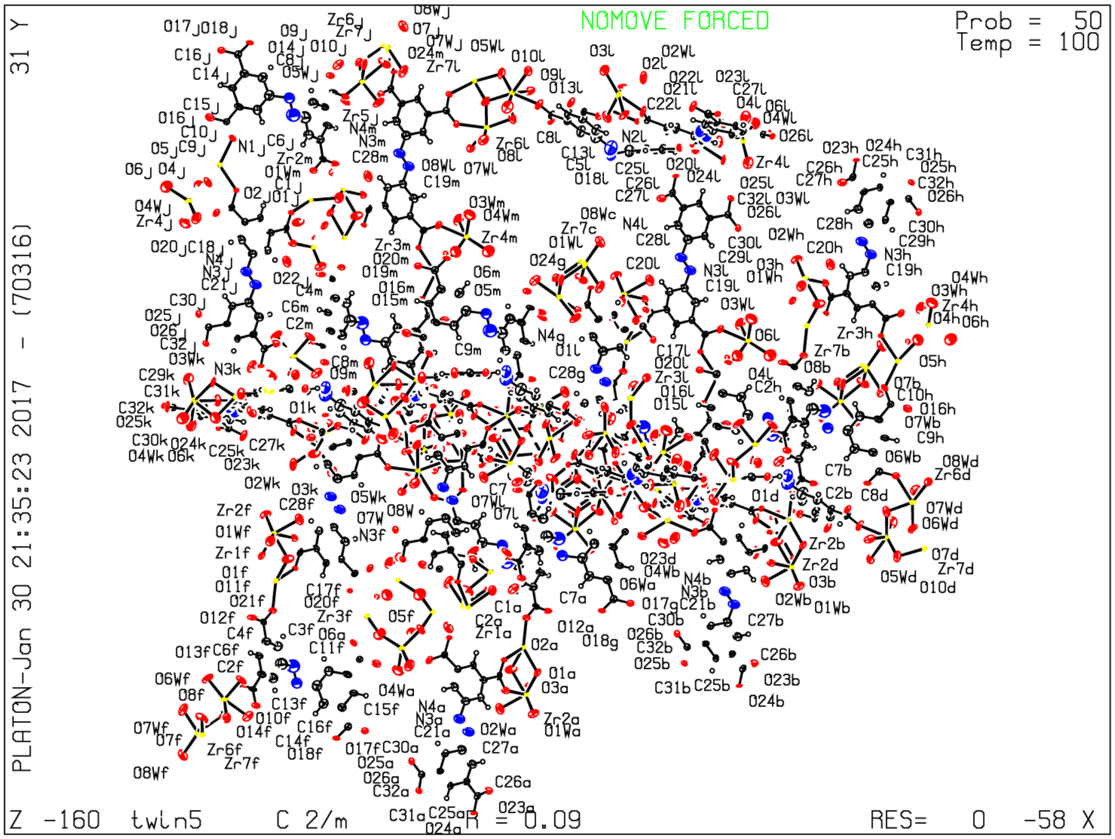

Supplement: Supplementary file 7 — Supplementary Data 4 [file 41467_2018_4152_MOESM7_ESM.pdf]
