# Supplementary material for: Topologically guided tuning of Zr-MOF pore structures for highly selective separation of C6 alkane isomers
Source: Nat Commun. 2018 May 1;9:1745. doi: 10.1038/s41467-018-04152-5 (PMC5931593; doi:10.1038/s41467-018-04152-5)

checkCIF/PLATON report

Structure factors have been supplied for datablock(s) hwg7-35_a_sq

THIS REPORT IS FOR GUIDANCE ONLY. IF USED AS PART OF A REVIEW PROCEDURE FOR PUBLICATION, IT SHOULD NOT REPLACE THE EXPERTISE OF AN EXPERIENCED CRYSTALLOGRAPHIC REFEREE.

No syntax errors found.   CIF dictionary   Interpreting this report

Datablock: hwg7-35_a_sq

```
Bond precision:  C-C = 0.0233 A          Wavelength=0.77490

Cell:           a=25.2379(14)     b=27.7529(16)     c=15.2344(10)
                alpha=90          beta=90           gamma=90
Temperature:    100 K

                Calculated                  Reported
Volume          10670.6(11)                 10670.6(11)
Space group     I m m a                     I m m a
Hall group      -I 2b 2                     -I 2b 2
Moiety formula  C14 H6 O16 Zr3 [+ solvent]  ?
Sum formula     C14 H6 O16 Zr3 [+ solvent]  C14 H17 O16 Zr3
Mr              703.85                      714.93
Dx,g cm-3       0.876                       0.890
Z               8                           8
Mu (mm-1)       0.756                       0.757
F000            2704.0                      2792.0
F000'           2662.40
h,k,lmax        25,27,15                    25,27,15
Nref            3032                        2978
Tmin,Tmax       0.938,0.970                 0.585,0.970
Tmin'           0.860

Correction method= # Reported T Limits: Tmin=0.585 Tmax=0.970
AbsCorr = MULTI-SCAN

Data completeness= 0.982          Theta(max)= 22.990

R(reflections)= 0.1484( 1872)     wR2(reflections)= 0.4420( 2978)

S = 3.131                 Npar= 172
```

The following ALERTS were generated. Each ALERT has the format
test-name_ALERT_alert-type_alert-level.
Click on the hyperlinks for more details of the test.

Alert level A

THETM01_ALERT_3_A The value of sine(theta_max)/wavelength is less than 0.550
Calculated sin(theta_max)/wavelength = 0.5040

Author Response: The data were cut at 1.0 Angstroms above above the I/sigma(I) < 2 meaning on average the data was not observed.

PLAT973_ALERT_2_A Check Calcd Positive Residual Density on Zr1 4.97 eA-3

Author Response: The crystals all showed strong diffuse scattering in the form of diffuse Bragg peaks and scattering between them. As the model does not account for this type of disorder this is believe to be the source of the high electron density on the Zr.

PLAT973_ALERT_2_A Check Calcd Positive Residual Density on Zr2 3.73 eA-3

Author Response: The crystals all showed strong diffuse scattering in the form of diffuse Bragg peaks and scattering between them. As the model does not account for this type of disorder this is believe to be the source of the high electron density on the Zr.

Alert level B

PLAT084_ALERT_3_B High wR2 Value (i.e. > 0.25) .... 0.44 Report

Author Response: The crystals all showed strong diffuse scattering in the form of diffuse Bragg peaks and scattering between them. As the model does not account for this type of disorder this is results in a high wR2.

PLAT094_ALERT_2_B Ratio of Maximum / Minimum Residual Density .... 4.12 Report

Author Response: The crystals all showed strong diffuse scattering in the form of diffuse Bragg peaks and scattering between them. As the model does not account for this type of disorder

PLAT097_ALERT_2_B Large Reported Max. (Positive) Residual Density 4.55 eA-3

Author Response: The crystals all showed strong diffuse scattering in the form of diffuse Bragg peaks and scattering between them. As the model does not account for this type of disorder this is believe to be the source of the high electron density on the Zr.

```
PLAT342_ALERT_3_B Low Bond Precision on  C-C Bonds ...............     0.02333 Ang.
```

Author Response: The crystals all showed strong diffuse scattering in the form of diffuse Bragg peaks and scattering between them. As the model does not account for this type of disorder this is results in low bond precision.

Alert level C

```
CHEMW03_ALERT_2_C The ratio of given/expected molecular weight as
            calculated from the _atom_site* data lies outside
            the range 0.99 <> 1.01
           From the CIF: _cell_formula_units_Z                    8
           From the CIF: _chemical_formula_weight           714.93
           TEST: Calculate formula weight from _atom_site_*
           atom     mass    num     sum
           C        12.01   14.00  168.15
           H         1.01    6.00    6.05
           O        16.00   16.00  255.98
           Zr       91.22    3.00  273.67
           Calculated formula weight             703.86
DIFMX02_ALERT_1_C  The maximum difference density is > 0.1*ZMAX*0.75
            The relevant atom site should be identified.
GOODF01_ALERT_2_C  The least squares goodness of fit parameter lies
            outside the range 0.80 <> 2.00
            Goodness of fit given =      3.131
PLAT082_ALERT_2_C High R1 Value ..................................       0.15 Report
PLAT087_ALERT_2_C Unsatisfactory S value (Too High) ..............       3.13 Check
PLAT234_ALERT_4_C Large Hirshfeld Difference Zr1    --  O1      ..       0.19 Ang.
PLAT234_ALERT_4_C Large Hirshfeld Difference Zr2    --  O3      ..       0.16 Ang.
PLAT241_ALERT_2_C High   'MainMol' Ueq as Compared to Neighbors of         O3 Check
PLAT242_ALERT_2_C Low    'MainMol' Ueq as Compared to Neighbors of        Zr1 Check
PLAT242_ALERT_2_C Low    'MainMol' Ueq as Compared to Neighbors of        Zr2 Check
PLAT250_ALERT_2_C Large U3/U1 Ratio for Average U(i,j) Tensor ....        3.7 Note
PLAT905_ALERT_3_C Negative K value in the Analysis of Variance ...    -15.234 Report
PLAT905_ALERT_3_C Negative K value in the Analysis of Variance ...     -0.296 Report
PLAT911_ALERT_3_C Missing # FCF Refl Between THmin & STh/L=  0.504         50 Report
PLAT975_ALERT_2_C Check Calcd Residual Density  0.56A From      O3       1.20 eA-3
PLAT976_ALERT_2_C Check Calcd Residual Density  0.97A From      O2      -1.07 eA-3
PLAT978_ALERT_2_C Number C-C Bonds with Positive Residual Density.          0 Info
```

Alert level G

```
FORMU01_ALERT_2_G  There is a discrepancy between the atom counts in the
            _chemical_formula_sum and the formula from the _atom_site* data.
            Atom count from _chemical_formula_sum:C14 H17 O16 Zr3
            Atom count from the _atom_site data:  C14 H6 O16 Zr3
ABSMU01_ALERT_1_G  Calculation of _exptl_absorpt_correction_mu
                not performed for this radiation type.
CELLZ01_ALERT_1_G Difference between formula and atom_site contents detected.
CELLZ01_ALERT_1_G WARNING: H atoms missing from atom site list. Is this intentional?
           From the CIF: _cell_formula_units_Z    8
           From the CIF: _chemical_formula_sum  C14 H17 O16 Zr3
           TEST: Compare cell contents of formula and atom_site data

           atom    Z*formula  cif sites diff
           C        112.00    112.00    0.00
           H        136.00     48.00   88.00
```

```
          O        128.00    128.00    0.00
          Zr        24.00     24.00    0.00
PLAT003_ALERT_2_G Number of Uiso or Uij Restrained non-H Atoms ...         21 Report
PLAT004_ALERT_5_G Polymeric Structure Found with Maximum Dimension          3 Info
PLAT041_ALERT_1_G Calc. and Reported SumFormula    Strings  Differ     Please Check
PLAT068_ALERT_1_G Reported F000 Differs from Calcd (or Missing)...     Please Check
PLAT174_ALERT_4_G The CIF-Embedded .res File Contains FLAT Records          1 Report
PLAT178_ALERT_4_G The CIF-Embedded .res File Contains SIMU Records          1 Report
PLAT186_ALERT_4_G The CIF-Embedded .res File Contains ISOR Records          1 Report
PLAT187_ALERT_4_G The CIF-Embedded .res File Contains RIGU Records          1 Report
PLAT300_ALERT_4_G Atom Site Occupancy of C6      is Constrained at        0.5 Check
PLAT300_ALERT_4_G Atom Site Occupancy of C7      is Constrained at        0.5 Check
PLAT300_ALERT_4_G Atom Site Occupancy of C8      is Constrained at        0.5 Check
PLAT300_ALERT_4_G Atom Site Occupancy of H7      is Constrained at        0.5 Check
PLAT301_ALERT_3_G Main Residue  Disorder ..............(Resd  1)..         9% Note
PLAT606_ALERT_4_G VERY LARGE Solvent Accessible VOID(S) in Structure        ! Info
PLAT764_ALERT_4_G Overcomplete CIF Bond List Detected (Rep/Expd) .       1.41 Ratio
PLAT860_ALERT_3_G Number of Least-Squares Restraints .............        275 Note
PLAT869_ALERT_4_G ALERTS Related to the use of SQUEEZE Suppressed           ! Info
PLAT909_ALERT_3_G Percentage of I>2sig(I) Data at Theta(Max) Still        44% Note
PLAT910_ALERT_3_G Missing # of FCF Reflection(s) Below Theta(Min).          4 Note
PLAT913_ALERT_3_G Missing # of Very Strong Reflections in FCF ....          3 Note
PLAT933_ALERT_2_G Number of OMIT Records in Embedded .res File ...         19 Note
```

---

3 **ALERT level A** = Most likely a serious problem - resolve or explain
4 **ALERT level B** = A potentially serious problem, consider carefully
17 **ALERT level C** = Check. Ensure it is not caused by an omission or oversight
25 **ALERT level G** = General information/check it is not something unexpected

6 ALERT type 1 CIF construction/syntax error, inconsistent or missing data
18 ALERT type 2 Indicator that the structure model may be wrong or deficient
11 ALERT type 3 Indicator that the structure quality may be low
13 ALERT type 4 Improvement, methodology, query or suggestion
1 ALERT type 5 Informative message, check

It is advisable to attempt to resolve as many as possible of the alerts in all categories. Often the minor alerts point to easily fixed oversights, errors and omissions in your CIF or refinement strategy, so attention to these fine details can be worthwhile. In order to resolve some of the more serious problems it may be necessary to carry out additional measurements or structure refinements. However, the purpose of your study may justify the reported deviations and the more serious of these should normally be commented upon in the discussion or experimental section of a paper or in the "special_details" fields of the CIF. checkCIF was carefully designed to identify outliers and unusual parameters, but every test has its limitations and alerts that are not important in a particular case may appear. Conversely, the absence of alerts does not guarantee there are no aspects of the results needing attention. It is up to the individual to critically assess their own results and, if necessary, seek expert advice.

Publication of your CIF in IUCr journals

A basic structural check has been run on your CIF. These basic checks will be run on all CIFs submitted for publication in IUCr journals (*Acta Crystallographica*, *Journal of Applied Crystallography*, *Journal of Synchrotron Radiation*); however, if you intend to submit to *Acta Crystallographica Section C* or *E* or *IUCrData*, you should make sure that full publication checks are run on the final version of your CIF prior to submission.

Publication of your CIF in other journals

Please refer to the *Notes for Authors* of the relevant journal for any special instructions relating to CIF submission.

PLATON version of 13/08/2017; check.def file version of 27/07/2017

Datablock hwg7-35_a_sq - ellipsoid plot

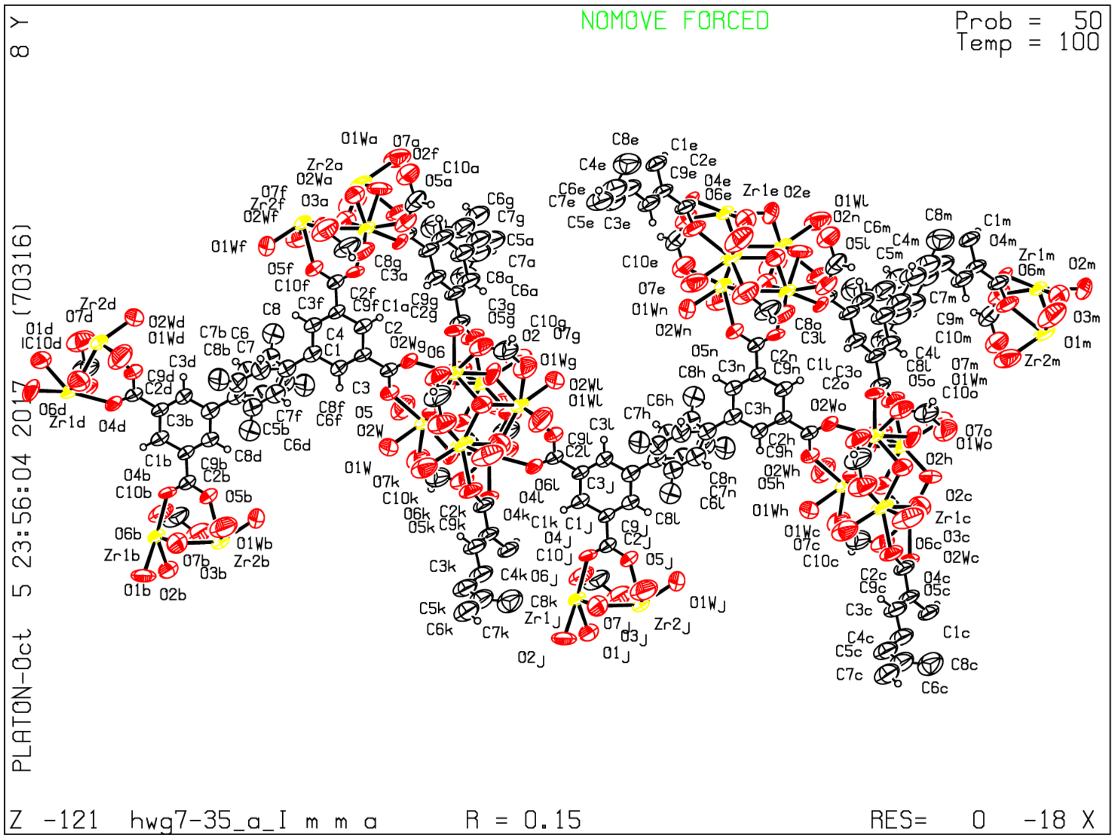

Supplement: Supplementary file 9 — Supplementary Data 6 [file 41467_2018_4152_MOESM9_ESM.pdf]
